# Homogeneous environmental selection mainly determines the denitrifying bacterial community in intensive aquaculture water

**DOI:** 10.3389/fmicb.2023.1280450

**Published:** 2023-11-02

**Authors:** Xiafei Zheng, Zhongneng Yan, Chenxi Zhao, Lin He, Zhihua Lin, Minhai Liu

**Affiliations:** ^1^Ninghai Institute of Mariculture Breeding and Seed Industry, Zhejiang Wanli University, Ningbo, China; ^2^Zhejiang Key Laboratory of Aquatic Germplasm Resource, College of Biological and Environmental Sciences, Zhejiang Wanli University, Ningbo, China

**Keywords:** *napA*, *nosZ*, aerobic denitrification, nitrate, nitrous oxide, aquaculture water

## Abstract

Nitrate reduction by *napA* (encodes periplasmic nitrate reductase) bacteria and nitrous oxide reduction by *nosZ* (encodes nitrous oxide reductase) bacteria play important roles in nitrogen cycling and removal in intensive aquaculture systems. This study investigated the diversity, dynamics, drivers, and assembly mechanisms of total bacteria as well as *napA* and *nosZ* denitrifiers in intensive shrimp aquaculture ponds over a 100-day period. Alpha diversity of the total bacterial community increased significantly over time. In contrast, the alpha diversity of *napA* and *nosZ* bacteria remained relatively stable throughout the aquaculture process. The community structure changed markedly across all groups over the culture period. Total nitrogen, phosphate, total phosphorus, and silicate were identified as significant drivers of the denitrifying bacterial communities. Network analysis revealed complex co-occurrence patterns between total, *napA*, and *nosZ* bacteria which fluctuated over time. A null model approach showed that, unlike the total community dominated by stochastic factors, *napA* and *nosZ* bacteria were primarily governed by deterministic processes. The level of determinism increased with nutrient loading, suggesting the denitrifying community can be manipulated by bioaugmentation. The dominant genus *Ruegeria* may be a promising candidate for introducing targeted denitrifiers into aquaculture systems to improve nitrogen removal. Overall, this study provides important ecological insights into aerobic and nitrous oxide-reducing denitrifiers in intensive aquaculture, supporting strategies to optimize microbial community structure and function.

## Introduction

Aquaculture is a rapidly expanding industry that now provides nearly half of the world’s fish consumed by humans worldwide, surpassing capture fisheries (FAO, 2022). To meet the growing global demand for protein, the Food and Agriculture Organization predicts that global aquaculture production will reach 202 million tons by 2030 (FAO, 2022). However, aquaculture must be carefully managed to avoid negative impacts on fish and the environment. Excessive accumulation of nutrients in water can lead to diseases in aquatic animals (Gao et al., 2018; Deng et al., 2021), with eutrophication being a major environmental concern associated with the rapid development of aquaculture (Edwards, 2015). In aquaculture systems, only about 25% of the nitrogen from feed is converted into biomass of the target organism (Crab et al., 2007), while the remaining nutrients are converted to nitrogenous compounds in the water (Hargreaves, 1998). Bacteria play a central role in the nitrogen cycle, and denitrification is a key process in this cycle (Deng et al., 2020b). Denitrification is an important pathway for converting nitrate to gaseous products, involving the sequential reduction of nitrate (NO_3_^−^) to nitrite (NO_2_^−^), nitric oxide (NO), nitrous oxide (N_2_O), and dinitrogen (N_2_) (Seitzinger et al., 2006). This process is regulated by enzymes such as nitrate reductase (encoded by the *narG*/*napA* gene), nitrite reductase (encoded by the *nirS*/*nirK* gene), nitric oxide reductase (encoded by the *norB* gene), and nitrous oxide reductase (encoded by the *nosZ* gene). A comprehensive understanding of denitrification in aquaculture systems can provide valuable insights into water quality management, particularly for bioaugmentation approaches in intensive aquaculture.

Aerobic denitrification refers to the denitrification carried out by aerobic denitrifiers in the presence of oxygen, where oxygen and nitrate are simultaneously utilized as electron acceptors (Robertson and Kuenen, 1984; Yang et al., 2020). Periplasmic nitrate reductase (Nap) and membrane-bound nitrate reductase (Nar) are two prokaryotic nitrate reductases that play key roles in the first step of denitrification (Sparacino-Watkins et al., 2014). It has been suggested that Nap is insensitive to oxygen and thus could be expressed under aerobic conditions, whereas the activity of Nar might be repressed by the presence of O_2_ (Reyes et al., 1996). The *napA* gene, encoding Nap, is often used as a functional marker to identify aerobic denitrifiers (Zhu et al., 2012; Huang et al., 2015; Deng et al., 2020b; Ren et al., 2021). In intensive aquaculture, dissolved oxygen levels typically need to be above 4 mg/L to meet the oxygen requirements of the cultured organisms. As a result, aerobic denitrification has attracted considerable attention for its potential applications in aquaculture systems (Deng et al., 2020b, 2021; Shu et al., 2022). The *napA* gene has been detected at high abundance in recirculating aquaculture systems using real-time quantitative PCR (qPCR) (Deng et al., 2020b). Aerobic denitrifying bacteria have also been isolated from aquaculture water and used to remove nitrogen from aquaculture wastewater (Deng et al., 2021; Shu et al., 2022). However, denitrification under aerobic conditions often leads to incomplete reduction of nitrate, resulting in the production of the undesirable greenhouse gas N_2_O (Otte et al., 1996; Colliver and Stephenson, 2000). N_2_O is the third most important greenhouse gas after CH_4_ and CO_2_ with a global warming potential 298 times higher than CO_2_, and has been recognized as a major contributor to ozone depletion (Ravishankara et al., 2009). Microbial reduction of N_2_O to N_2_, catalyzed by the N_2_O reductase gene (*nosZ*), is the only known sink for N_2_O in the biosphere (Hallin et al., 2018). The *nosZ* gene was detected by real-time quantitative PCR (qPCR) in recirculating aquaculture systems (Chen et al., 2021) and aquaculture sediments (Fang et al., 2022). Overall, *napA* denitrifying bacteria and *nosZ* denitrifying bacteria have shown great potential for achieving complete nitrogen removal in intensive aquaculture water.

In order to manipulate the denitrification process in intensive aquaculture water, it is critical to investigate the presence and dynamics of denitrifying bacteria in these environments. Intensive aquaculture ponds are characterized by high nitrate levels and sufficient dissolved oxygen provided by aerators, which can potentially support the proliferation of aerobic denitrifying bacteria. Additionally, the presence of high nitrogen levels and suspended particles in intensive aquaculture ponds can create anaerobic microenvironments within the suspended particles, which can facilitate anaerobic denitrification (PÁEz-Osuna, 2001; Yan et al., 2019). Quantification of key denitrifying genes has been performed using quantitative polymerase chain reaction (qPCR) in the recirculating aquaculture systems (Deng et al., 2020a,b). However, the taxonomic diversity, composition, and dynamics of *napA* and *nosZ* denitrifying bacteria in the water of intensive aquaculture ponds are not yet well understood. In addition, understanding the underlying mechanisms governing community assembly can provide valuable insights for bioaugmentation strategies in intensive aquaculture. According to the niche-based theory of community assembly, environmental factors such as temperature, pH, dissolved oxygen, and salinity, as well as biological interactions such as predation, competition, and mutualism, determine the distribution and composition of species (Chave, 2004). In contrast, the neutral theory states that species dynamics are largely controlled by random processes such as birth, death, speciation, extinction, and immigration, and that all species are ecologically equivalent (Hubbell, 2001). It is generally accepted that both deterministic and stochastic processes simultaneously determine community assembly (Zhou and Ning, 2017; Ning et al., 2020). The degree of determinism in community assembly determines the predictability of the community and increases the potential for microbial community manipulation (Zheng et al., 2023a). Previous studies have shown that the assembly of denitrifying bacterial communities in bioreactors is primarily driven by stochastic processes (Wang D. et al., 2023), but more studies are needed to fully understand the community assembly mechanism of denitrifying bacteria in intensive aquaculture water.

The coastal intensive aquaculture pond, also known as a higher place pond, is a specific type of pond constructed with cement and lacking soil at the bottom, which distinguishes it from traditional aquaculture ponds. The microorganisms in the coastal intensive aquaculture pond initially originate from the source water and subsequently interact with the biotic and abiotic factors specific to these higher place ponds. The objectives of this study are to investigate (1) the diversity, structure, interaction, and taxonomy of *napA* and *nosZ* type bacteria in intensive aquaculture ponds, (2) the dynamics of denitrifying bacteria and their relationships with environmental parameters, and (3) the community assembly of denitrifying bacteria in intensive aquaculture ponds. We hypothesize that the diversity, composition, and interaction of aerobic denitrifying bacteria and nitrous oxide reducing bacteria exhibit dynamic patterns during aquaculture process and highly influenced by nutrient loading. Furthermore, we propose that the community assembly of these bacteria is influenced by determinism, which becomes more evident with increasing nutrient loading. To test our hypotheses, we will analyze the dynamic changes in community diversity and structure, as well as investigate the mechanisms underlying the community assembly of total, *napA*, and *nosZ* bacteria in the intensive aquaculture water of black tiger shrimp (*Penaeus monodon*) over a period of 100 days. Our findings have important implications for the bioaugmentation of denitrifiers in intensive aquaculture ponds for achieving complete nitrogen removal. By understanding the dynamics and composition of denitrifying bacterial communities, we can guide the targeted addition of specific denitrifying bacteria to enhance nitrogen removal efficiency in aquaculture water.

## Materials and methods

### Sampling ponds and procedure

The Yize Aquaculture Company, located on Shepan Island in China, served as our sampling site for this study. The source water used in the aquaculture system was initially pumped from a canal connected to Sanmen Bay and stored in two sterilized ponds. Microorganisms in the sterilized ponds were eliminated using quicklime. After settling for 3–5 days, the treated source water was then pumped into the culture ponds. These concrete culture ponds each had an area of 900 m^2^ and depth of 1.8 meters. The salinity of water in the ponds ranged from 14‰ to 20‰.

The aquaculture process of *Penaeus monodon* consisted of two stages. The first intermediary nursery stage lasted 30 days in four ponds. On day 10 after stocking, water replenishment was initiated by adding 15 cm every two days until normal levels were reached. Subsequently, 10% water exchange was performed every three days. After 30 days, shrimp were transferred to another eight grow-out ponds for the second stage. During this stage, 10% of the water was exchanged daily. Each pond typically yielded ~3,500 kg of shrimp over 100 days, and to achieve this yield, 7,000–8,750 kg of formulated feed was added. The formulated feed primarily consisted of 43% crude protein, 6% crude fat, 16% crude ash, 12% water, 1.2% total phosphorus, and 1–4% calcium.

In terms of sampling, during the first stage, water samples were collected from the four culture ponds, the source water, and the sterilized ponds on days 10, 20, and 30 after shrimp introduction. In the second stage, samples were collected from five culture ponds, source water, and sterilized ponds on days 40, 50, 60, 70, 80, and 100 after shrimp introduction. At each timepoint, 3–5 replicate samples were taken from each pond. In total, 59 samples were collected, comprising 41 samples from the culture ponds, 9 samples from the source water, and 9 samples from the sterilized ponds. The water samples were immediately transported to the laboratory at a temperature of 4°C within 10 min. Approximately 500 mL from each sample was filtered through 0.22 μm polycarbonate filters (47 mm diameter, Whatman) to collected bacteria. Filters were stored at −80°C until DNA extraction.

### Environmental variables analysis

A YSI EXO2 multi-parameter water quality probe was used to measure pH, temperature, dissolved oxygen, and salinity *in situ* (Yellow Springs Instruments, United States). Ammonia, nitrite, phosphate, total phosphorus, and silicate were measured according to the national standard method in China, “The Specifications for Oceanographic Survey Part 4: Survey of Chemical Parameters in Seawater” (GB/T 12763-2007). Nitrate was determined using the cadmium-copper column reduction method (Wood et al., 1967). Total nitrogen was determined by a combination of the high-pressure bomb method and the cadmium-copper column reduction method. A PHYTO-PAM II Phytoplankton Analyzer was used to estimate the chlorophyll a concentration (Walz, Germany). This method allows for the differentiation of phytoplankton groups based on the differences in their light-harvesting pigment antennae and the estimation of chlorophyll a concentrations of cyanobacteria, green algae, diatoms, and cryptophytes (Beutler et al., 2002).

### DNA extraction and amplicon sequencing

The PowerWater DNA Isolation Kit was used to extract the total microbial DNA from the filters (MO BIO laboratories, Carlsbad, United States). The *napA* gene was amplified with the primers *napA*F1 (CTGGACIATGGGYTTIAACCA) and *napA*R1 (CCTTCYTTYTCIACCCACAT) (Alcántara-Hernández et al., 2009). The *nosZ* gene was amplified with the primers *nosZ*2F (CGCRACGGCAASAAGGTSMSSGT) and *nosZ*2R (CAKRTGCAKSGCRTGGCAGAA). The 16S rRNA gene was amplified with the primers 338F (ACTCCTACGGGAGGCAGCA) and 806R (GGACTACHVGGGTWTCTAAT). The specific amplification procedures for the *napA*, *nosZ*, and 16S rRNA genes are described in detail in the Supplementary material of the study. The amplified fragments of the 16S rRNA, *nosZ*, and *napA* genes were then sequenced using the Illumina NovaSeq 6,000 PE250 platform by Guangdong Magigene Biotechnology (Guangzhou, China). The generated sequences have been deposited in the Sequence Read Archive (SRA) database at the National Center for Biotechnology Information (NCBI) under the BioProject ID PRJNA951088. The 16S rRNA gene sequences can be found under accession numbers SAMN34033797- SAMN34033855, the *nosZ* gene sequences under SAMN34036005- SAMN34036063, and the *napA* gene sequences under SAMN34036151- SAMN34036191.

### Bioinformatics analysis

The raw sequencing data were mapped to the corresponding sample barcodes, and the primers located at the beginning and end of the reads were trimmed to account for one mismatch. Paired end reads of sufficient length with at least 10 bp overlap were merged using the Usearch (version 11.0.667) fastq_mergepairs command with the default parameters (Edgar, 2010). Next, sequences with poor alignment and quality were filtered out using the Btrim program with version 0.2.0 (Kong, 2011). Specifically, sequences with a quality score of less than 20 within a window size of 4 were eliminated. The remaining high quality sequences were then subjected to further analysis. For the *nosZ* and *napA* genes, the FrameBot tool (version 1.2) on the FunGene pipeline of the Ribosomal Database Project (RDP) was utilized to examine the sequences for frame shifts (Wang et al., 2013). The sequences were then denoised and clustered into unique sequences (zero-distance operational taxonomic units; zOTU) by using the UNOISE algorithm implemented in Usearch (version 11.0.667). Meanwhile, the chimeric sequences were removed in the denoising procedure. To facilitate subsequent community analysis and statistical comparisons, all samples (16S rRNA, *nosZ*, and *napA*) were standardized to the same sequencing depth (37,827 sequences per sample) by resampling. The RDP v18 database was used for the taxonomic identification of 16S rRNA gene, while taxonomy annotations for *napA* and *nosZ* genes utilized databases provided by Lei Zhang from Logic Informatics Co., Ltd., Jinan, China. The *napA* and *nosZ* genes databases can be found on the GitHub.^1^

### Metacommunity diversity analysis

According to the dendrogram analysis, the 100-day culture period could be divided into three stages, namely stage 1 (10, 20, and 30 days), stage 2 (40, 50, and 60 days), and stage 3 (70, 80, and 100 days) (Supplementary Figure S7). The Rao quadratic entropy was used to quantify the contribution of local diversity and the difference among local diversity (Escalas et al., 2013). In brief, the metacommunity diversity over a period was expressed as the sum of the mean local community diversity, the intertemporal difference, and the mean intratemporal difference. As we divided the 100 days into three stages, the temporal diversity at each stage (γ_ecosystem_) was partitioned into the contribution of local diversity ( $\bar{\alpha }$ _LocalCommunity_), the diversity among different times (*β*_Intertemporal_), and the mean diversity among different local diversities at a given time ( $\bar{\beta }$ _Intratemporal_) with: γ_ecosystem_ = $\bar{\alpha }$ _LocalCommunity_ + *β*_Intertemporal +_ $\bar{\beta }$ _Intratemporal_.

### Microbial co-occurrence network construction and analysis

Co-occurrence networks were constructed for *napA* and *nosZ* denitrifiers separately, using samples from stage 1 to stage 3 of the aquaculture period. Additionally, a bipartite co-occurrence network was constructed to investigate the relationships between total bacteria and *napA* denitrifiers, total bacteria and *nosZ* denitrifiers, as well as between *napA* and *nosZ* denitrifiers. To ensure the reliability of the co-occurrence networks, only taxa that were observed in more than half of the samples were included. The network was constructed using Sparse Correlation for Compositional data (SparCC) with a threshold value of 0.6 and a significance level of 0.05. Network visualization and modularity measures were conducted with Gephi version 0.10. Network robustness refers to the proportion of remaining species in the network after random node removal. This calculation was calculated following the method proposed by Yuan et al. (2021). Microbial co-occurrence network construction and global property analysis were carried out using the integrated Network Analysis Pipeline (iNAP) (Feng et al., 2022).

### Microbial community assembly mechanism

The relative influences of community assembly processes were evaluated using a phylogenetic bin-based null model framework called iCAMP (Ning et al., 2020). First, the observed taxa were divided into different bins based on their phylogenetic signal. Second, ecological processes within each bin were quantified using phylogenetic diversity measured by the beta Net Relatedness Index (βNRI) and the taxonomic β-diversities measured by Raup–Crick metric (RC). Classification of the ecological process (heterogeneous selection, homogeneous selection, dispersal limitation, homogenizing dispersal, and drift.) was performed according to the iCAMP pipeline. Third, the proportion of each ecological process at the community level was then calculated by aggregating the percentages across all bins. Finally, linear regression analysis was performed to examine the relationship between environmental parameters and the microbial community determinism ratios to identify significant factors driving the denitrifying bacterial community.

### Statistical analysis

The statistical significance of the alpha diversity of the bacterial community throughout the aquaculture process was assessed using the Kruskal-Wallis test. The mean alpha diversity index between the shrimp pond and the source water was compared using a two-tailed Student’s *t*-test. The variation in the structure of the bacterial community was examined using principal coordinates analysis (PCoA) based on the Bray-Curtis index. The significance of the community structure was tested using permutational multivariate analysis of variance (PERMANOVA). The bacterial dynamic pattern was analyzed by calculating Pearson’s correlation between the date and the relative abundance of taxa, with *p*-values adjusted using the false discovery rate (FDR). The relationships between microbial community and environmental parameters were analyzed by Mantel test. Additionally, the relationships among the environmental parameters were analyzed using Pearson’s correlation. All statistical significance was considered at *p* < 0.05.

## Results and discussion

### Variations of environmental parameters in the water

The temperature, which ranged from 26 to 34°C, and salinity, which ranged from 14 to 21‰, showed similar dynamic trends across pond water, sterilized pond, and source water (Supplementary Figure S1). The pH remained relatively stable in the pond water, ranging between 7.6 and 8.6, while pH fluctuated more sharply in the sterilized pond and source water, ranging from 5.4–9.4 and 6.0–7.7, respectively (Supplementary Figure S1). In addition, dissolved oxygen levels were maintained at adequate levels by aeration in the pond water, averaging 7.2 ± 1.2 mg/L (Supplementary Figure S1). The ammonium fluctuated dramatically, peaking on day 50 (2.3 ± 0.1 mg/L), followed by a decline on days 60 and 70 (0.3 ± 0.2 mg/L), and a subsequent increase on day 100 (1.7 ± 0.9 mg/L, Supplementary Figure S2). Simultaneously, nitrite and nitrate levels increased from day 10 (nitrite: 0.003 ± 0.002 mg/L, nitrate: 0.01 ± 0.01 mg/L) to day 100 (nitrite: 3.7 ± 0.9 mg/L, nitrate: 2.2 ± 0.9 mg/L). Inorganic nitrogen showed a drop at day 80, likely due to water exchange around that time, while total nitrogen continued increasing throughout the 100-day period (Supplementary Figure S2). Similarly, phosphate and total phosphorus exhibited a sharp increase in the first month, with phosphate decreasing slightly during day 60 and day 80. However, total phosphorus remained relatively stable at a high level after day 30 (Supplementary Figure S3). Our results indicated that, despite fluctuations in inorganic nitrogen and phosphate after day 30, total nitrogen and total phosphorus levels remained consistently high from day 30 to day 100. This suggests organic nitrogen and organic phosphorus accumulation during culture may serve as an essential nutrient source (Gál et al., 2016), similar to findings in polyculture ponds (Zheng et al., 2017). Interestingly, our data showed silicates remained at lower levels in pond water (3.3 ± 2.5 μmol/L) compared to sterilized water (31.5 ± 10.1 μmol/L) and source water (42.2 ± 20.9 μmol/L), indicating silicate limitation in intensive aquaculture water (Supplementary Figure S3). Similarly, diatoms abundance decreased during the first month and remained at low levels thereafter (Supplementary Figure S4). In contrast, green algae increased, suggesting potential competition between diatoms and green algae in intensive aquaculture water (Sommer, 1994). Despite cyanobacteria presence in the source water, their abundance remained low in pond water and only increased around day 80 (Supplementary Figure S4).

### Community diversity and structure of total bacteria and denitrifying bacteria

**Figure 1:**
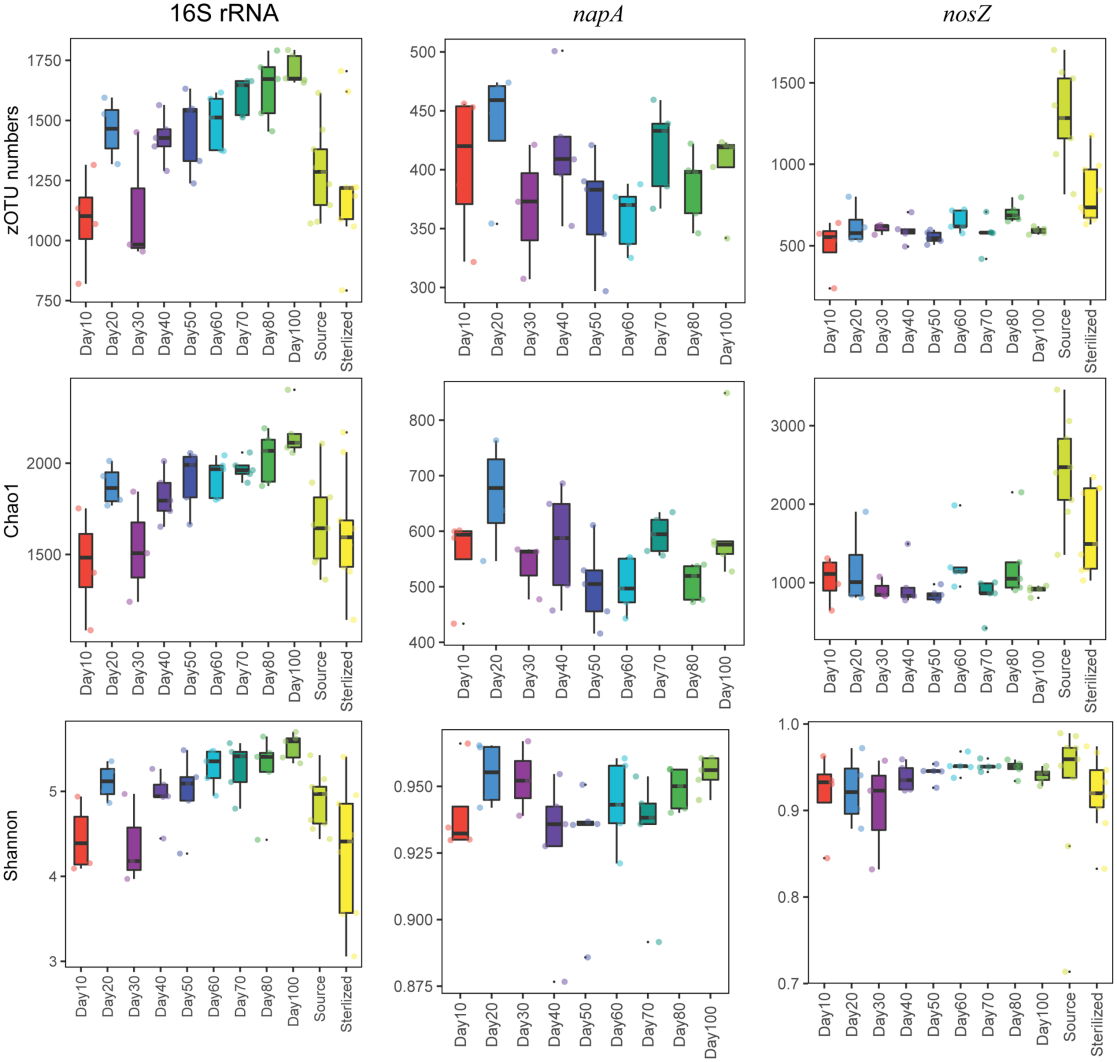
The dynamics of zOTU numbers, Chao1 index, and Shannon index of the total, *napA*, and *nosZ* bacterial communities.

**Figure 2:**
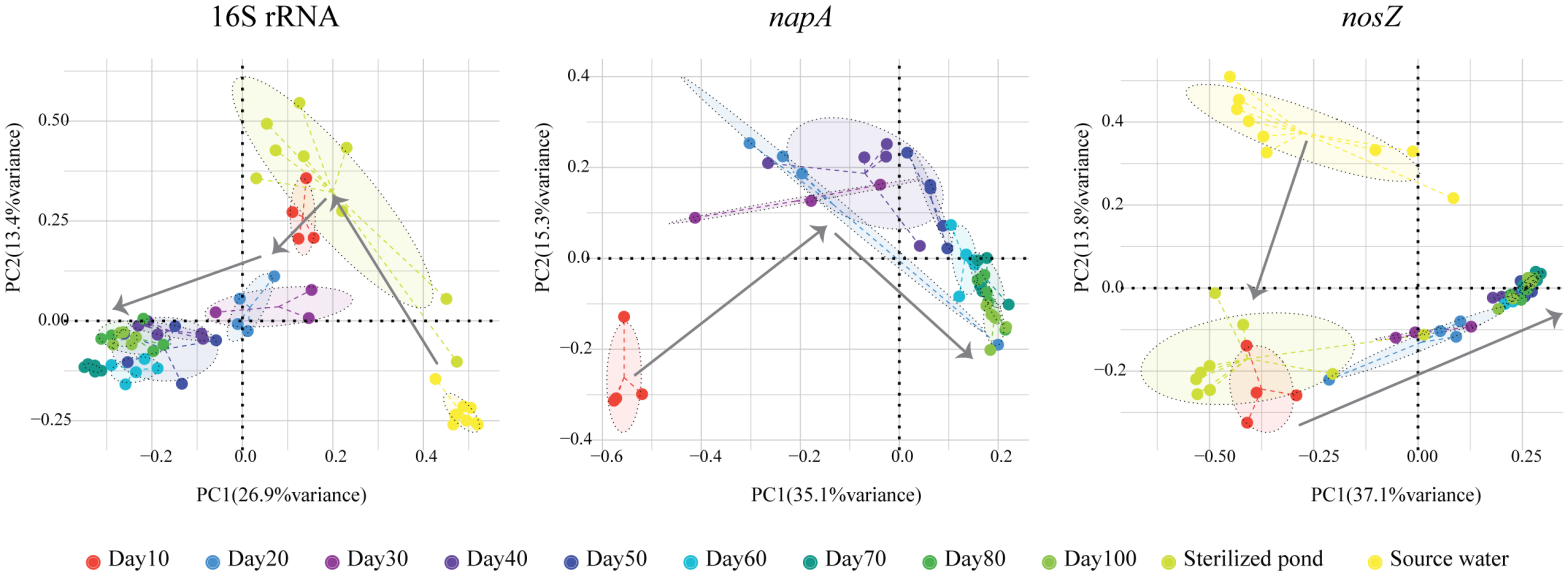
Principal Coordinates Analysis (PCoA) of total, *napA* and *nosZ* bacterial communities in aquaculture water over a period of 100 days.

We found that the number of zOTUs, Chao1, and the Shannon index all increased significantly over time for the total bacterial community (*p* < 0.05, Kruskal Wallis test, Figure 1). The mean number of zOTUs for the total bacterial community was higher in the shrimp pond than in the source water, but there was no significant difference in Chao1 and Shannon index between them (*p* > 0.05, *t*-test, Supplementary Figure S5). When comparing the Shannon index between the shrimp pond and the source water from stage 1 to stage 3, no significant differences were observed in stage 1 and stage 2 (*p* > 0.05, *t*-test, Supplementary Figure S6), but the Shannon index was significantly higher in the shrimp pond than in the source water in stage 3 (*p* < 0.05, *t*-test, Supplementary Figure S6). Additionally, the structure of total bacterial community was significantly different over the 100 days (*p* < 0.05, PERMANOVA, Figure 2).

For the *napA* bacterial community, none of the water samples from the source water or the sterilized ponds contained the *napA* gene. The alpha diversity indices were not significantly influenced by the sampling date (*p* > 0.05, Kruskal Wallis test, Figure 1), but the community structure was significantly different over the 100 days (*p* < 0.05, PERMANOVA, Figure 2). Previous studies have reported the presence of aerobic denitrifiers in various environments such as activated sludge, wastewater, sulfate treatment plants, soil, and freshwater sediment (Patureau et al., 2000; Ji et al., 2015). Our results showed that *napA*-type denitrifying bacteria were abundant in the water of coastal intensive aquaculture ponds, but were absent in the source water and sterilized ponds. Similar to other aerobic denitrifiers found in anthropogenic systems (Ji et al., 2015), our results indicate that intensive aquaculture provides an ideal environment for the enrichment of *napA*-type denitrifying bacteria. In intensive aquaculture ponds, the high protein feed load provides sufficient nitrogen sources, and intermittent aeration promotes the growth of aerobic denitrifying bacteria. In contrast to the widespread occurrence of aerobic denitrifiers in natural environments, our results suggest that natural coastal water might either lack aerobic denitrifiers or their abundance is too low to detect. However, it should be noted that we did not examine aerobic denitrifiers in the sediment of the bay, and further research is needed to assess the diversity of *napA*-type denitrifiers in coastal sediment.

For the *nosZ* bacterial community, the alpha diversity indices were not significantly influenced by the sampling date (*p* > 0.05, Kruskal Wallis test, Figure 1), but the community structure was significantly influenced by the sampling date (*p* < 0.05, PERMANOVA, Figure 2). The alpha diversity of the *nosZ* bacterial community was significantly lower in the pond water compared to the source water (*p* < 0.05, *t*-test, Supplementary Figure S5). In contrast to the total bacterial diversity, the Shannon index was significantly lower in the pond water than in the source water at all stages (*p* < 0.05, *t*-test, Supplementary Figure S6). Denitrification is typically known to occur under facultative anaerobic or microanoxic conditions (Kuypers et al., 2018). Although the dissolved oxygen concentration in the aquaculture system is high, microanoxic zones exist in the inner regions of suspended particles (Yan et al., 2019), providing a suitable niche for *nosZ*-type denitrifiers. In contrast to *napA*-type denitrifying bacteria, we observed lower alpha community diversity indices for *nosZ*-type denitrifying bacteria in the intensive aquaculture water compared to the source water. Our results suggest that only a fraction of *nosZ* denitrifying bacteria from the source water can survive and adapt in the aquaculture water due to the unfavorable conditions for denitrification in the presence of excess oxygen (Chen and Strous, 2013).

We quantified local, intertemporal, and intratemporal community diversity to assess changes in different aspects of diversity during bacterial community succession from stage 1 to 3 (Table 1). The percentage of $\bar{\alpha }$ _LocalCommunity_ was relatively stable, and the $\bar{\beta }$ _Intratemporal_ slightly increased in the total bacterial community from stage 1 to 3 (Table 1). However, the $\bar{\alpha }$ _LocalCommunity_ increased, while *β*_Intertemporal_, and $\bar{\beta }$ _Intratemporal_ decreased in the *napA*- and *nosZ*-type bacterial communities from stage 1 to 3. Our findings revealed that the functional groups of denitrifying bacteria underwent an assembly process leading to more homogeneous across communities over time. A potential explanation is that some shared ecological pressures selected for certain *napA*- and *nosZ* bacterial lineages across all sites as time progressed.

**Table 1:** Multiscales hierarchical partitioning of bacteria community diversity.

Bacteria type	Diversity	Stage 1	Stage 2	Stage 3
Total	GammaReg%	100	100	100
	AlphaLoc%	47.58	57.93	47.49
	BetaInter%	27.85	15.07	20.13
	BetaIntra%	24.58	27.00	32.38
*napA*	GammaReg%	100	100	100
	AlphaLoc%	41.46	78.00	81.94
	BetaInter%	35.93	6.17	4.97
	BetaIntra%	22.62	15.83	13.09
*nosZ*	GammaReg%	100	100	100
	AlphaLoc%	40.12	87.26	88.55
	BetaInter%	43.84	3.49	4.96
	BetaIntra%	16.04	9.24	6.48

The temporal diversity at each stage (γ_ecosystem_) was partitioned into the contribution of local diversity ( $\bar{\alpha }$ _LocalCommunity_), the diversity among different times (β_Intertemporal_), and the mean diversity among different local diversity at a certain time ( $\bar{\beta }$ _Intratemporal_) with: γ_ecosystem_ = $\bar{\alpha }$ _LocalCommunity_ + β_Intertemporal +_ $\bar{\beta }$ _Intratemporal_.

### Bacterial community composition and dynamic pattern

The relative abundance of the *napA* gene has been frequently determined in recirculating aquaculture systems using qPCR (Deng et al., 2020a,b), but its taxonomic diversity in coastal intensive aquaculture ponds has not yet been studied. To investigate the taxonomic composition of the bacterial communities, we analyzed their composition at the high rank (phylum or class) and low rank (genus) levels (Supplementary Figures S8–S10). For the total bacterial community, Proteobacteria, Bacteroidetes, and Actinobacteria were the dominant bacterial phyla, accounting for about 92% percentage of the community (Supplementary Figure S8). At the genus level, *Ruegeria* was the most dominant genus (10.0%), followed by *Maribacter* (3.2%), *Tenacibaculum* (2.5%), *Hyphomonas* (1.2%), *Flavobacterium* (1.1%), *Marivita* (1.1%), *Motilimonas* (0.8%), *Erythrobacter* (0.7%), and *Robiginitalea* (0.6%) (Supplementary Figure S8). For *napA*-type denitrifier, Proteobacteria accounted for 99.9% percentage of the bacterial community, with 84.3% belonging to Alphaproteobacteria and 14.3% belonging to Gammaproteobacteria (Supplementary Figure S9). At the genus level, the top 10 genera were *Roseibium* (14.8%), *Palleronia* (13.4%), *Ruegeria* (10.0%), *Noviherbaspirillum* (6.7%), *Zhengella* (4.7%), *Jhaorihella* (3.3%), *Motilimonas* (3.2%), *Aliiroseo*var*ius* (2.5%), and *Ferrimonas* (1.4%) (Supplementary Figure S9). For *nosZ*-type bacteria, all zOTUs belonged to Proteobacteria, with 99.4% belonging to Alphaproteobacteria and only 0.4% belonging to Gammaproteobacteria (Supplementary Figure S10). At the genus level, *Ruegeria* (18.2%), *Sedimentitalea* (17.8%), *JL08* (5.2%), *Ruegeria_B* (4.7%), *Marimonas* (4.2%), *Marivita* (2.3%) were the genera representing more than 1% percentage of the bacterial community (Supplementary Figure S10). Proteobacteria was the most dominant phylum in both the total and denitrifying bacterial communities, accounting for approximately 99% of the denitrifying bacteria carrying either *napA* or *nosZ* genes. *Ruegeria* and *Motilimonas* were considered dominant genera in both total bacteria and *napA*-type denitrifying bacteria, while *Ruegeria* and *Marivita* were dominant genera in both total bacteria and *nosZ*-type denitrifying bacteria. The dominant aerobic denitrifiers in our study differed from those in recirculating aquaculture systems (Deng et al., 2020a,b). Notably, commonly isolated aerobic denitrifiers from aquaculture environments, such as *Bacillus* (Gao et al., 2018), *Marinobacter* (Liu et al., 2016), *Vibrio* (Ren et al., 2021), and *Pseudomonas* (Deng et al., 2014; Hong et al., 2020; Wang et al., 2020; Wang A. et al., 2023), were not dominant in our samples. This finding suggests that the prevailing aerobic denitrifying bacteria may not hold dominance in intensive aquaculture water as previously believed. Interestingly, *Ruegeria* was the dominant genus in the total, *napA*, and *nosZ* bacterial communities which is rarely reported and used in applications. *Ruegeria* is a common denitrifier carrying a complete gene set for denitrification in coastal environments (Lin et al., 2022; Meng et al., 2022). Therefore, future applications of denitrifying bacteria may target the isolation of the *Ruegeria* geneus.

Our analysis revealed significant positive or negative trends with the aquaculture date for many taxa, ranging from the phylum to the genus level, within the total bacterial community (Supplementary Table S1). Specifically, the relative abundance of *Vibrio*, a potentially pathogenic bacterium, increased significantly during the aquaculture process, while *Bacillus*, often considered a probiotic bacterium, decreased significantly. However, there were fewer taxa showing significant dynamic trends in *napA*-type bacteria, and even fewer in *nosZ*-type bacteria, during the aquaculture period. We found that the relative abundance of Proteobacteria in the total bacterial community increased during the aquaculture period. However, the relative abundance of Gammaproteobacteria increased while that of Alphaproteobacteria decreased during the aquaculture process in the *napA*-type bacteria. Previous studies have found that isolated aerobic denitrifiers in the natural and managed ecosystems belong mainly to Betaproteobacteria, Alphaproteobacteria, and Gammaproteobacteria classes (Patureau et al., 2000; Ji et al., 2015). Similarly, a molecular-based survey found that *napA* denitrifiers in aquatic reservoirs are primarily affiliated with these classes (Zhou et al., 2019). In our study, we found that intensive aquaculture water predominantly contained aerobic denitrifiers belonging to the Alphaproteobacteria and Gammaproteobacteria classes, with the absence of Betaproteobacteria. This suggests a distinct distribution pattern of aerobic denitrifying bacteria in intensive aquaculture water compared to other systems. Furthermore, our results indicated a preference for Gammaproteobacteria and an inhibition of Alphaproteobacteria within the aquaculture water as the aquaculture operation continued. Our results show that many bacteria had obvious dynamic patterns with the aquaculture process, but denitrifying bacteria, particularly the *nosZ* denitrifiers, were less influenced by the aquaculture date.

### Microbial co-occurrence network of denitrifying bacteria

**Figure 3:**
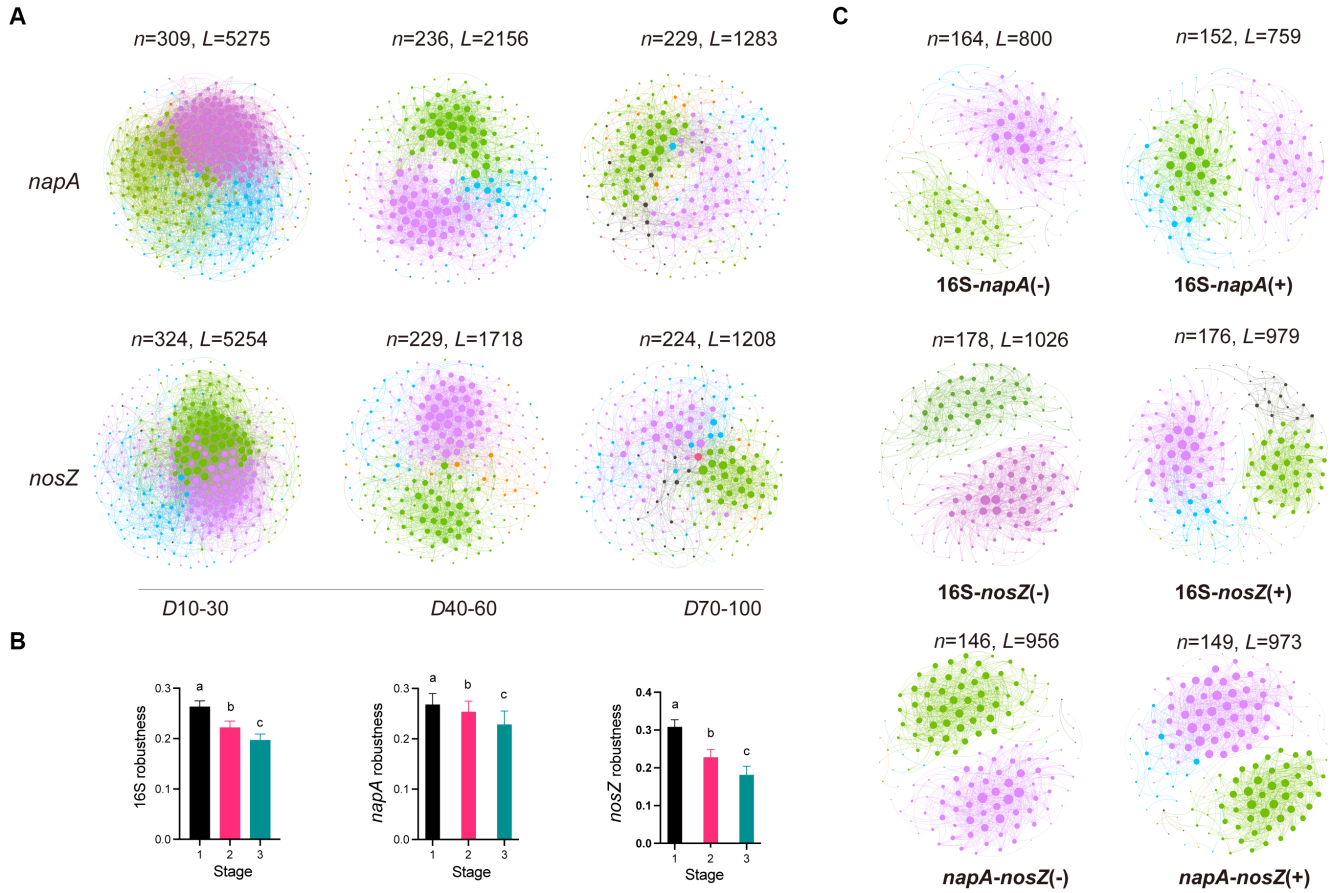
Microbial co-occurrence network and dynamics in the *napA* and *nosZ* denitrifying bacterial community. **(A)** Co-occurrence network and its robustness **(B)** in the total bacterial communities, *napA* bacterial communities, and *nosZ* bacterial communities during three stages: stage 1 (day 10–30), stage 2 (day 40–60), and stage 3 (day 70–100). **(C)** Bipartite co-occurrence network between total bacteria and *napA* denitrifiers, total bacteria and *nosZ* bacteria, as well as between *napA* and *nosZ* bacteria. The positive and negative bipartite networks are indicated by “+” and “−”, respectively. N represents the number of nodes, while L represents the number of edges. The size of a node is proportional to its degree. Modules are colored randomly.

The dynamics of the co-occurrence networks in total bacteria, *napA* denitrifiers and *nosZ* denitrifiers show both similarities and contrasts over time (Figures 3A,B; Supplementary Table S2). While the number of nodes in the total bacterial network exhibits an increasing trend, the number of nodes in the *napA* and *nosZ* denitrifier networks decreases. This suggests a potential loss of diversity within the active denitrifier communities. Moreover, all three groups show a decrease in the number of links, indicating a decrease in overall connectivity. The average degree, clustering coefficient, and path distance also decrease, suggesting a reduction in overall network connectivity and complexity. Additionally, we observed a decrease in the robustness of the co-occurrence networks in all groups, indicating that network stability decreased during the aquaculture period. However, the maximal betweenness, centralization of betweenness, and eigenvector centrality increase over time indicating the growing importance of specific nodes with multiple interactions to other denitrifers.

To further our analysis, we investigated the bipartite co-occurrence networks between total bacteria and two denitrifier groups, namely *napA* denitrifiers and *nosZ* denitrifiers, as well as between the two denitrifier groups themselves (Figure 3C). The results revealed a variety of positive and negative relationships among total bacteria, *napA* and *nosZ* denitrifiers, and a clear modularity was observed in these bipartite networks. Most of the bipartite networks had 2–4 major modules, and these modules were not fixed but displayed fluctuations in their abundance over time (Supplementary Figure S11). Throughout the aquaculture period, most of the modules demonstrated either an increasing or decreasing trend. These results showed that the co-occurrence patterns of denitrifiers were not static, but they changed over time in intensive aquaculture water. Bacteria within the same module exhibit high cooperation in nutrient metabolism, and the switch between modules allow them to adapt effectively to changing aquaculture water conditions and fully utilize nitrogen and carbon (Huang et al., 2020). We further found that *Maribacter* and *Ruegeria* were dominant taxa present in both the total bacterial community and the *nosZ* denitrifiers in the co-occurrence network (Supplementary Figure S13). This could be attributed to the high salinity tolerance and robust denitrification capabilities of *Maribacter* and *Ruegeria* in saline conditions (Li et al., 2013; Lin et al., 2022). Moreover, *Maribacter* is known to utilize lipid compounds, a primary ingredient in formulated feed, and its secondary metabolites may benefit denitrifers (Bonin et al., 2015). Additionally, *Ruegeria* exhibits a particle-associated life style, contributing to the release of nutrient from suspended particles, which in turn benefit denitrifers in the aquaculture water (Sonnenschein et al., 2017).

### Relationships between bacterial community and environmental parameters

**Figure 4:**
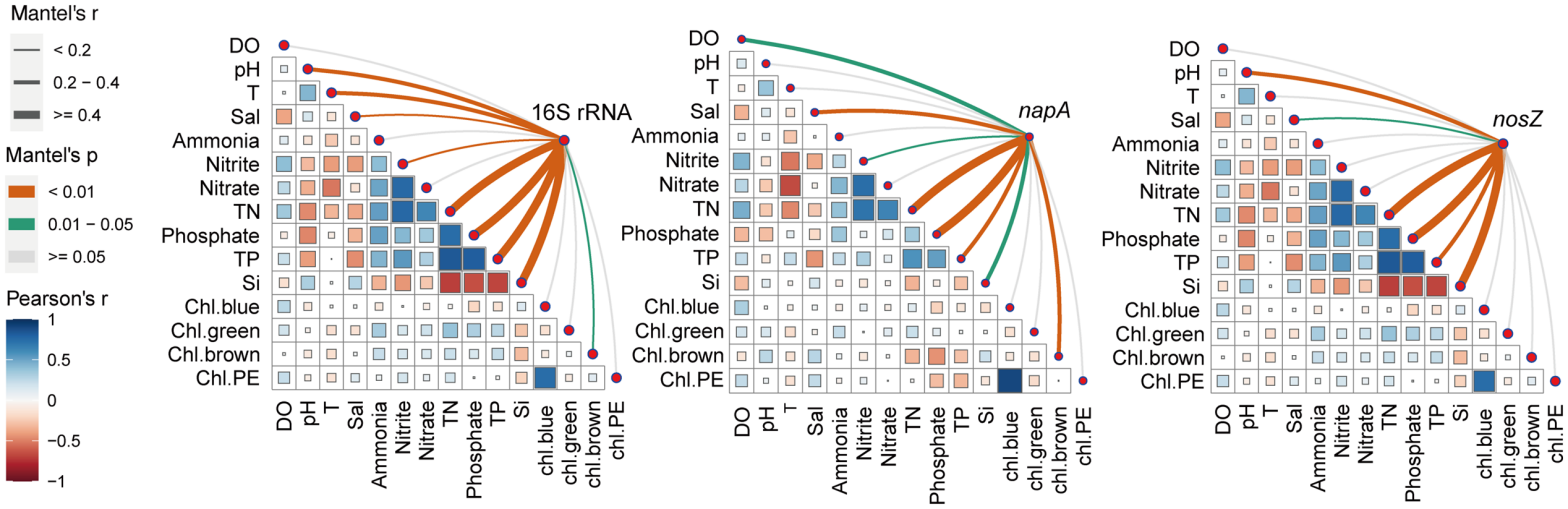
Correlations between environmental factors and microbial community structure. The total, *napA*-type, and *nosZ*-type bacterial community structure based on Bray–Curtis distance is related to each environmental factor by partial Mantel test. Line width corresponds to the partial Mantel’s r statistic, and line color indicates the statistical significance based on 999 permutations. Pairwise comparisons of environmental factors are also shown, with a color gradient indicating Pearson’s correlation coefficient. DO, dissolved oxygen; T, temperature; Sal, salinity; TN, total nitrogen; TP, total phosphorus; Chl.bule, chl.green, chl.brown, and chl.PE represent the chlorophyll a concentrations of cyanobacteria, green algae, diatoms, and cryptophytes, respectively.

First, we analyzed the correlated relationships among environmental parameters (Figure 4). We found that TN had a positive correlation with ammonia, nitrite, nitrate, and TP. TP was positively correlated with phosphate and TN. TN and TP had negative correlations with silicate. The positive correlation between TN and TP in aquaculture systems is attributed to the high concentration of suspended organic particles in the water, which serve as a main nutrient resource (Zheng et al., 2017). At the beginning of the culture, the source water has a high silicate concentration, which gradually decreases throughout the culture period, while nitrogen and phosphorus concentrations continue to increase, explaining the negative relationship between TN and TP with silicate. Secondly, based on the Mantel results, we found that TN, phosphate, TP, silicate, and salinity significantly influenced the total, *napA*, and *nosZ* type bacteria (Figure 4). In addition, the total bacterial community was influenced by pH, temperature, nitrite, and diatom. The *napA* type bacteria were influenced by DO, nitrite, and diatom. The *nosZ* type-bacteria were significantly influenced by pH. We further discovered that the primary environmental factors influencing denitrifying bacteria varied slightly between different stages. In stage 1, the significant increase in total nitrogen, pH, and phosphate, as well as the sudden decrease in silicate and diatoms compared to source and sterilized water, made them the key factors driving the bacterial community (Supplementary Figure S15). In stage 2, pH, temperature, and nitrogen were the main drivers of the denitrifying bacterial community, while the total bacterial community was also affected by silicate (Supplementary Figure S15). In stage 3, an apparent increase in salinity was the main factor driving the dynamics of the bacterial community (Supplementary Figure S15). Nitrate, which serves as the terminal electron acceptor during denitrification, plays a significant role in shaping the denitrifying bacterial community in aquaculture, consistent with previous research (Niu et al., 2023). Moreover, salinity has been frequently reported to have a high association with denitrifiers and generally has a negative effect on them (Deng et al., 2017; von Ahnen et al., 2019). Interestingly, our study also revealed a close relationship between silicate and the bacterial community, particularly the denitrifying bacterial community, which has been rarely reported. Xia et al. (2023) have demonstrated that the denitrifying bacteria community is not solely influenced by changes in physical and chemical factors but also exhibits a significant correlation with algae density. Bacteria and phytoplankton in aquaculture ponds maintain close mutualistic relationships (Zheng et al., 2023b). Algae play a crucial role by providing organic carbon, which serves as an electron donor for denitrifiers during the denitrification process (Xia et al., 2023). Silicate and diatom have close relationships since diatoms utilize silicate to build their cell wall. Diatoms are rich sources of vitamins, essential amino acids, minerals, essential fatty acids, and carotenoid pigments for the shrimp (Ju et al., 2009). Our study demonstrated that silicate was rapidly consumed after larval shrimp were introduced into the ponds, displaying an opposite trend compared to nitrogen and phosphorus levels. However, we acknowledge that denitrifying bacterial communities can be affected by additional factors beyond those examined here. For example, the availability of organic carbon sources (Grießmeier and Gescher, 2018), the carbon to nitrogen ratios (Deng et al., 2020a), the presence of heavy metals (Chen et al., 2020). Further studies should pay more attention to the roles of silicate, carbon, and heavy metal in bacterial community dynamic in coastal aquaculture water.

### Community assembly mechanism

**Figure 5:**
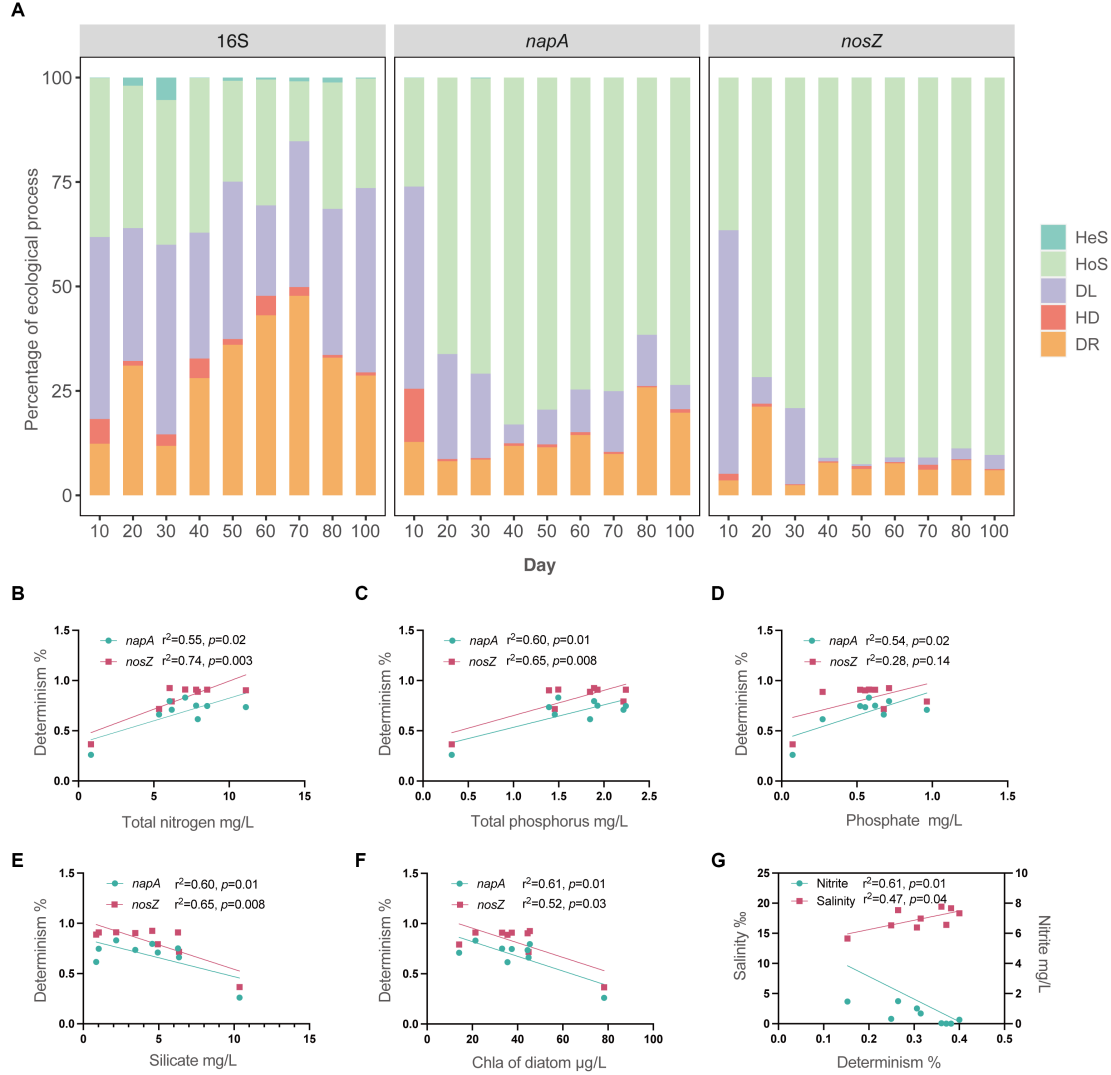
Microbial community assembly and environmental drivers. **(A)** Detailed assembly processes governing the microbial community of total bacteria, *napA* denitrifier, and *nosZ* denitrifier during the 100-day period. Hes, heterogeneous selection; HoS, homogeneous selection; DL, dispersal limitation; HD, homogenizing dispersal; DR, drift. **(B–F)** Linear relationships between environmental factors and the percentage of determinism in *napA* and *nosZ* denitrifying communities. **(G)** Linear relationships between environmental factors and the percentage of determinism in the total bacterial community.

Our results revealed that the total bacterial community was predominantly governed by stochasticity throughout the entire aquaculture period, with percentages ranging from 60 to 85% (Figure 5A). Specifically, homogeneous selection, dispersal limitation, and drift were the primary influences on the total bacterial community, which is consistent with previous research highlighting the impact of stochasticity on bacterial communities in aquaculture water (Hou et al., 2021). In contrast, both *napA* and *nosZ* denitrifying bacterial communities were predominantly determined by deterministic factors (61–92%, Figure 5A), except for day 10, which exhibited a high percentage of stochasticity (*napA*: 74%; *nosZ*: 63%, Figure 5A). Specifically, the high stochasticity on day 10 was attributed to dispersal limitation, after which the denitrifying bacterial community was governed by homogeneous selection from day 20 to day 100 (Figure 5A). The community assembly mechanism of denitrifying bacterial communities varies in previous studies. Some studies have shown that the denitrifying bacterial communities in river sediments (Zhang et al., 2021), epiphytic biofilms of a lake (Li et al., 2022), laboratory microcosms (Liu and Sheng, 2021), and agricultural soils (Wang W. et al., 2022) are mainly governed by stochasticity, with dispersal limitation as the main ecological process. Other studies showed that denitrifying bacterial communities in bioreactor (Wang D. et al., 2022, 2023), corpse degradation water (Yu et al., 2021), and along elevation gradients of mountain (Kou et al., 2021) were mainly governed by determinism rather than stochasticity. These studies found determinism as the dominant role all emphasized environmental filtering in regulating denitrifying communities in terms of heterogeneous selection or homogeneous selection. In this study, we also found *napA* and *nosZ* denitrifying bacteria in intensive aquaculture water were mainly governed by determinism. Aquaculture pond and bioreactors share a similarity in terms of the continuous addition of nutrients. Intensive aquaculture ponds can be viewed as large-scale bioreactors with high nutrient loading, creating an environment that promotes the enrichment of denitrifiers.

Furthermore, we assessed the linear relationships between available environmental parameters and the percentage of determinism in microbial communities (Figures 5B–G). Notably, we found significant associations between the percentage of determinism in *napA* and *nosZ* bacterial communities and total nitrogen, total phosphorus, phosphate, silicate, and diatom chlorophyll *a* (Figures 5B–G). These environmental factors were also identified as the key factors associated with bacterial community structure. Particularly, an increase in total nitrogen, total phosphorus, and phosphate, as well as a decrease in silicate and diatom chlorophyll *a*, will lead to an increase in determinism within the denitrifying bacterial community. The total bacterial community was found to be primarily governed by stochasticity. However, we were able to identify salinity and nitrite as the only factors that had significant relationships with the limited determinism observed in the total bacterial community. Denitrifying bacterial community is not only associated to nitrogen but also influenced by carbon (Wang et al., 2021). Total nitrogen and total phosphorus are comprehensive indicators, and the increase of total nitrogen and total phosphorus in the aquaculture water due to formulated feeding supplementation includes the increase of both nitrogen and carbon (Hargreaves, 1998; Crab et al., 2007). Thus total nitrogen and total phosphorus became the prominent factor contributing the determinism of denitrifying bacterial community. The difference between denitrifying bacteria and total bacteria may attribute to their different phylogenetic diversity. The denitrifying bacteria mainly belong to Proteobacteria, however the total bacteria fall into 22 phyla. The denitrifying bacteria as nitrogen functional bacteria have similar nutrient strategy and accordingly more synchronized in a corresponding manner.

Microbial bioaugmentation involves introducing specific microbial strains or consortia into contaminated environments to enhance pollutant degradation (El Fantroussi and Agathos, 2005). However, whether supplementing aquaculture systems with exogenous bacteria can improve water quality has been debated, due to uncertainties about survival and activity of introduced microbes (Hong et al., 2021). Our findings demonstrate that diverse assemblages of denitrifying bacteria harboring *napA* and *nosZ* genes already exist in intensive aquaculture ponds. Furthermore, increasing nutrient loads, especially nitrogen, appeared to strengthen the homogeneous selection of denitrifier communities via deterministic assembly shaped by the pond environment. These findings suggest that high nutrient levels can facilitate the adaptation and colonization of supplemented denitrifying bacteria in intensive aquaculture water. Thus, our results provide a theoretical basis supporting the supplementation of nitrogen-cycling bacteria to manipulate microbial communities and improve water quality in intensive aquaculture water. Successful denitrifier bioaugmentation has consistently occurred in high nitrogen environments, indicating strong determinism governing the denitrifying bacterial community (Hong et al., 2020; Wang A. et al., 2023). Overall, our study suggests that high nitrogen loading in intensive aquaculture water will likely benefit denitrifier bioaugmentation by enhancing homogeneous environmental selection within their bacterial community.

## Conclusion

This study revealed novel insights into the diversity, dynamics, and assembly mechanisms of denitrifying bacterial communities in intensive shrimp aquaculture ponds over a period of 100 days. In contrast to the total bacterial community, the alpha diversity and composition of *napA* and *nosZ* bacteria remained relatively stable throughout the aquaculture process. Nutrient parameters including nitrogen, phosphorus, and silicates were identified as key drivers shaping the denitrifying communities. Unlike the stochastic assembly of the total bacteria, deterministic factors governed the assembly of *napA* and *nosZ* denitrifiers, enhanced by nutrient loading. The dominance of determinism for these functional groups suggests denitrifier bioaugmentation could effectively promote targeted community structure and nitrogen removal. *Ruegeria* emerged as an abundant genus across the denitrifier groups, representing a potential candidate for introducing aerobic and nitrous oxide-reducing strains into aquaculture systems. By elucidating the ecology of denitrifying bacteria, this study provides a foundation to guide microbial management strategies for improving water quality in intensive aquaculture. Further research should investigate the outcomes of deliberately manipulating the denitrifier community structure through bioaugmentation in aquaculture ponds.

## Data availability statement

The datasets presented in this study can be found in online repositories. The names of the repository/repositories and accession number(s) can be found in the article/Supplementary material.

## Author contributions

XZ: Data curation, Formal analysis, Investigation, Methodology, Visualization, Writing – original draft, Writing – review & editing. ZY: Investigation, Methodology, Writing – review & editing. CZ: Investigation, Methodology, Writing – review & editing. LH: Resources, Writing – review & editing. ZL: Funding acquisition, Project administration, Supervision, Writing – review & editing. ML: Conceptualization, Funding acquisition, Project administration, Writing – review & editing.

## Footnotes

^1^ https://github.com/zhengxf88/napA-nosZ-database
