# Supplementary material for: Homogeneous environmental selection mainly determines the denitrifying bacterial community in intensive aquaculture water
Source: Front Microbiol. 2023 Nov 2;14:1280450. doi: 10.3389/fmicb.2023.1280450 (PMC10653326; doi:10.3389/fmicb.2023.1280450)
Supplement: Supplementary file 1 [file Data_Sheet_1.docx]

**Homogeneous environmental selection mainly determines the denitrifying bacterial community in intensive aquaculture water**

Xiafei Zheng^a, b^, Zhongneng Yan^a, b^, Chenxi Zhao^a, b^, Lin He^a, b^, Zhihua Lin^a, b^, Minhai Liu^a, b^

^a^ Ninghai Institute of Mariculture Breeding and Seed Industry, Zhejiang Wanli University, Ningbo, China

^b^ Zhejiang Key Laboratory of Aquatic Germplasm Resource, College of Biological and Environmental Sciences, Zhejiang Wanli University, Ningbo, China

Corresponding author: Minhai Liu, E-mail: minhailiu@zwu.edu.cn; Zhihua Lin, E-mail: zhihua9988@126.com

**The PCR program of *napA*, *nosZ*, and 16S rRNA genes**

The *napA* gene fragement amplification protocol consisted of an initial denaturalization at 96°C for 3 mins, followed by 30 cycles at 96°C (1 min), 52°C (30 s) and 72°C (30 s), with a final extension step at 72°C (10 min). The *nosZ* gene fragement amplification protocol of consisted of an initial denaturalization at 94°C for 5 mins, followed by 35 cycles at 94°C (30 s), 63°C (45 s) and 72°C (30 s), with a final extension step at 72°C (8 min). The 16S rRNA gene amplification protocol consisted of an initial denaturalization at 94°C for 5 mins, followed by 30 cycles at 94°C (30 s), 53°C (30 s) and 72°C (30 s), with a final extension step at 72°C (8 min).

Table S1 The bacterial dynamic pattern across the aquaculture process. The bacterial taxa that had significant correlations (r > or < 0.6, *p* < 0.05) with date were represented. *P* value was adjusted using the false discovery rate (FDR).

| Bacterial type | Taxa rank | Taxa | Correlation r |
| --- | --- | --- | --- |
| 16S | Phylum | Acidobacteria | 0.8 |
| 16S | Phylum | Rhodothermaeota | 0.8 |
| 16S | Phylum | Deinococcus_Thermus | 0.8 |
| 16S | Phylum | Firmicutes | -0.7 |
| 16S | Phylum | Actinobacteria | -0.6 |
| 16S | Phylum | Proteobacteria | 0.6 |
| 16S | Class | Rhodothermia | 0.8 |
| 16S | Class | Acidobacteria_Gp3 | 0.7 |
| 16S | Class | Actinobacteria | -0.6 |
| 16S | Class | Acidobacteria_Gp10 | 0.6 |
| 16S | Class | Bacilli | -0.6 |
| 16S | Class | Blastocatellia | 0.6 |
| 16S | Class | Clostridia | -0.6 |
| *napA* | Class | Gammaproteobacteria | 0.7 |
| *napA* | Class | Alphaproteobacteria | -0.6 |
| 16S | Order | Rhodothermales | 0.8 |
| 16S | Order | Vibrionales | 0.7 |
| 16S | Order | Parvularculales | 0.7 |
| 16S | Order | Micrococcales | -0.7 |
| 16S | Order | Bacillales | -0.6 |
| 16S | Order | Mycobacteriales | 0.6 |
| 16S | Order | Clostridiales | -0.6 |
| *napA* | Order | Xanthomonadales | 0.7 |
| *napA* | Order | Rhodospirillales | -0.6 |
| *napA* | Order | Burkholderiales | 0.6 |
| *nosZ* | Order | Rhizobiales | -0.6 |
| 16S | Family | Rubricoccaceae | 0.8 |
| 16S | Family | Vibrionaceae | 0.7 |
| 16S | Family | Parvularculaceae | 0.7 |
| 16S | Family | Microbacteriaceae | -0.7 |
| 16S | Family | Pseudoalteromonadaceae | 0.7 |
| 16S | Family | Salisaetaceae | 0.6 |
| 16S | Family | Bacillaceae_1 | -0.6 |
| 16S | Family | Polyangiaceae | 0.6 |
| 16S | Family | Mycobacteriaceae | 0.6 |
| 16S | Family | Cyclobacteriaceae | -0.6 |
| 16S | Family | Phyllobacteriaceae | 0.6 |
| 16S | Family | Clostridiales_Incertae_Sedis_XII | -0.6 |
| 16S | Family | Alteromonadales_incertae_sedis | 0.6 |
| *napA* | Family | SZUA_36 | 0.7 |
| *napA* | Family | Burkholderiaceae | 0.6 |
| *napA* | Family | Alteromonadaceae | -0.6 |
| *nosZ* | Family | Rhizobiales | -0.6 |
| 16S | Genus | *Rubrivirga* | 0.8 |
| 16S | Genus | *Vibrio* | 0.7 |
| 16S | Genus | *Gp3* | 0.7 |
| 16S | Genus | *Pseudoalteromonas* | 0.7 |
| 16S | Genus | *Paracoccus* | 0.6 |
| 16S | Genus | *Bacillus* | -0.6 |
| 16S | Genus | *Mycobacterium* | 0.6 |
| 16S | Genus | *Devosia* | -0.6 |
| 16S | Genus | *Fusibacter* | -0.6 |
| 16S | Genus | *Maribacter* | 0.6 |
| 16S | Genus | *Motilimonas* | 0.6 |
| 16S | Genus | *Mariniphaga* | -0.6 |
| 16S | Genus | *Stappia* | -0.6 |
| 16S | Genus | *Algoriphagus* | -0.6 |
| *napA* | Genus | *W260* | 0.7 |
| *napA* | Genus | *Achromobacter* | -0.6 |
| *napA* | Genus | *Noviherbaspirillum* | 0.6 |
| *napA* | Genus | *Algibacillus* | -0.6 |
| *napA* | Genus | *Rhodovulum* | -0.6 |
| *napA* | Genus | *Saccharophagus* | 0.6 |
| *nosZ* | Genus | *Roseovarius* | -0.6 |
| *nosZ* | Genus | *JL08* | -0.6 |

Table S2 Topological parameters of microbial co-occurrence network

|  | 16S | | | *napA* | | | *nosZ* | | |
| --- | --- | --- | --- | --- | --- | --- | --- | --- | --- |
|  | day10-30 | day40-60 | day70-100 | day10-30 | day40-60 | day70-100 | day10-30 | day40-60 | day70-100 |
| Total nodes | 827 | 888 | 1035 | 309 | 236 | 229 | 334 | 229 | 224 |
| Total links | 16342 | 13462 | 9926 | 5275 | 2156 | 1283 | 5254 | 1718 | 1208 |
| R square of power-law | 0.87 | 0.94 | 0.93 | 0.51 | 0.80 | 0.84 | 0.83 | 0.91 | 0.92 |
| Average degree (avgK) | 40 | 30 | 19 | 34 | 18 | 11 | 31 | 15 | 11 |
| Average clustering coefficient (avgCC) | 0.46 | 0.45 | 0.36 | 0.51 | 0.50 | 0.40 | 0.51 | 0.51 | 0.41 |
| Average path distance (GD) | 2.73 | 3.08 | 3.28 | 2.46 | 2.74 | 3.10 | 2.70 | 2.86 | 3.21 |
| Geodesic efficiency (E) | 0.41 | 0.37 | 0.34 | 0.47 | 0.42 | 0.37 | 0.44 | 0.41 | 0.36 |
| Harmonic geodesic distance (HD) | 2.45 | 2.73 | 2.96 | 2.13 | 2.36 | 2.67 | 2.28 | 2.47 | 2.74 |
| Maximal degree | 170 | 212 | 147 | 108 | 55 | 51 | 116 | 55 | 49 |
| Centralization of degree (CD) | 0.16 | 0.20 | 0.12 | 0.24 | 0.16 | 0.17 | 0.25 | 0.18 | 0.17 |
| Maximal betweenness | 6453 | 9210 | 15733 | 1191 | 1708 | 1747 | 1635 | 1851 | 2070 |
| Centralization of betweenness (CB) | 0.02 | 0.02 | 0.03 | 0.02 | 0.06 | 0.06 | 0.02 | 0.06 | 0.07 |
| Maximal stress centrality | 252686 | 349834 | 385119 | 30145 | 30241 | 18067 | 48309 | 28434 | 20896 |
| Centralization of stress centrality (CS) | 0.65 | 0.81 | 0.67 | 0.51 | 0.98 | 0.61 | 0.72 | 0.99 | 0.74 |
| Centralization of eigenvector centrality (CE) | 0.83 | 0.88 | 0.91 | 0.74 | 0.76 | 0.85 | 0.75 | 0.81 | 0.84 |
| Centralization of closeness centrality (CCL) | 0.23 | 0.08 | 0.08 | 0.26 | 0.02 | 0.02 | 0.04 | 0.03 | 0.25 |
| Density (D) | 0.05 | 0.03 | 0.02 | 0.11 | 0.08 | 0.05 | 0.09 | 0.07 | 0.05 |
| Transitivity (Trans) | 0.41 | 0.46 | 0.33 | 0.54 | 0.52 | 0.41 | 0.55 | 0.53 | 0.42 |
| Efficiency | 0.95 | 0.97 | 0.98 | 0.89 | 0.92 | 0.95 | 0.91 | 0.93 | 0.96 |
| Module number | 6 | 13 | 15 | 5 | 7 | 11 | 10 | 7 | 11 |
| modularity | 0.30 | 0.31 | 0.38 | 0.30 | 0.42 | 0.38 | 0.25 | 0.42 | 0.37 |

**Figure legend**

Figure S1 Variation of temperature, pH, dissolved oxygen, and salinity in pond water, sterilized water, and source water through the culture period.

Figure S2 Variation of ammonia, nitrite, nitrate, and total nirogen in pond water, sterilized water, and source water through the culture period.

Figure S3 Variation of phosphate, total phosphorus, and silicate in pond water, sterilized water, and source water through the culture period.

Figure S4 Variation of the diatom, green algae, cyanobacteria, and cryptophytes silicate in pond water, sterilized water, and source water through the culture period.

Figure S5 Comparison of zOTU numbers, chao1 index, Shannon index between shrimp pond water and source water.

Figure S6 Comparison of Shannon index between shrimp pond water and source water from stage 1 to stage 3.

Figure S7 Dendrogram analysis of total bacteria, *napA* and *nosZ* bacterial community based on Bray-Curtis dissimilarity.

Figure S8 Bacterial community composition determined by the 16S rRNA gene. (a) at the phylum level. (b) at the genus level (only top 10 genera were shown).

Figure S9 Bacterial community composition determined by the *napA* gene. (a) at the class level. (b) at the genus level (only top 10 genera were shown).

Figure S10 Bacterial community composition determined by the *nosZ* gene. (a) at the class level. (b) at the genus level (only top 10 genera were shown).

Figure S11 Relative abundance of bacteria in major modules of the bipartite co-occurrence network from day 10 to day 100.

Figure S12 Species counts in the co-occurrence network of total bacteria and *napA* denitrifiers. Unclassified taxa were not shown.

Figure S13 Species counts in the co-occurrence network of total bacteria and *nosZ* denitrifiers. Unclassified taxa were not shown.

Figure S14 Species counts in the co-occurrence network of *napA* denitrifiers and *napA* denitrifiers. Unclassified taxa were not shown.

Figure S15 Correlations between environmental factors and microbial community structure in stage 1, stage 2, and stage 3, respectively. The total, napA-type, and nosZ-type bacterial community structure based on Bray–Curtis distance is related to each environmental factor by partial Mantel test. Line width corresponds to the partial Mantel’s r statistic, and line color indicates the statistical significance based on 999 permutations. Pairwise comparisons of environmental factors are also shown, with a color gradient indicating Pearson’s correlation coefficient. DO: dissolved oxygen; T: temperature; Sal: salinity; TN: total nitrogen; TP: total phosphorus; Chl.bule, chl.green, chl.brown, and chl.PE represent the chlorophyll a concentrations of cyanobacteria, green algae, diatoms, and cryptophytes, respectively.


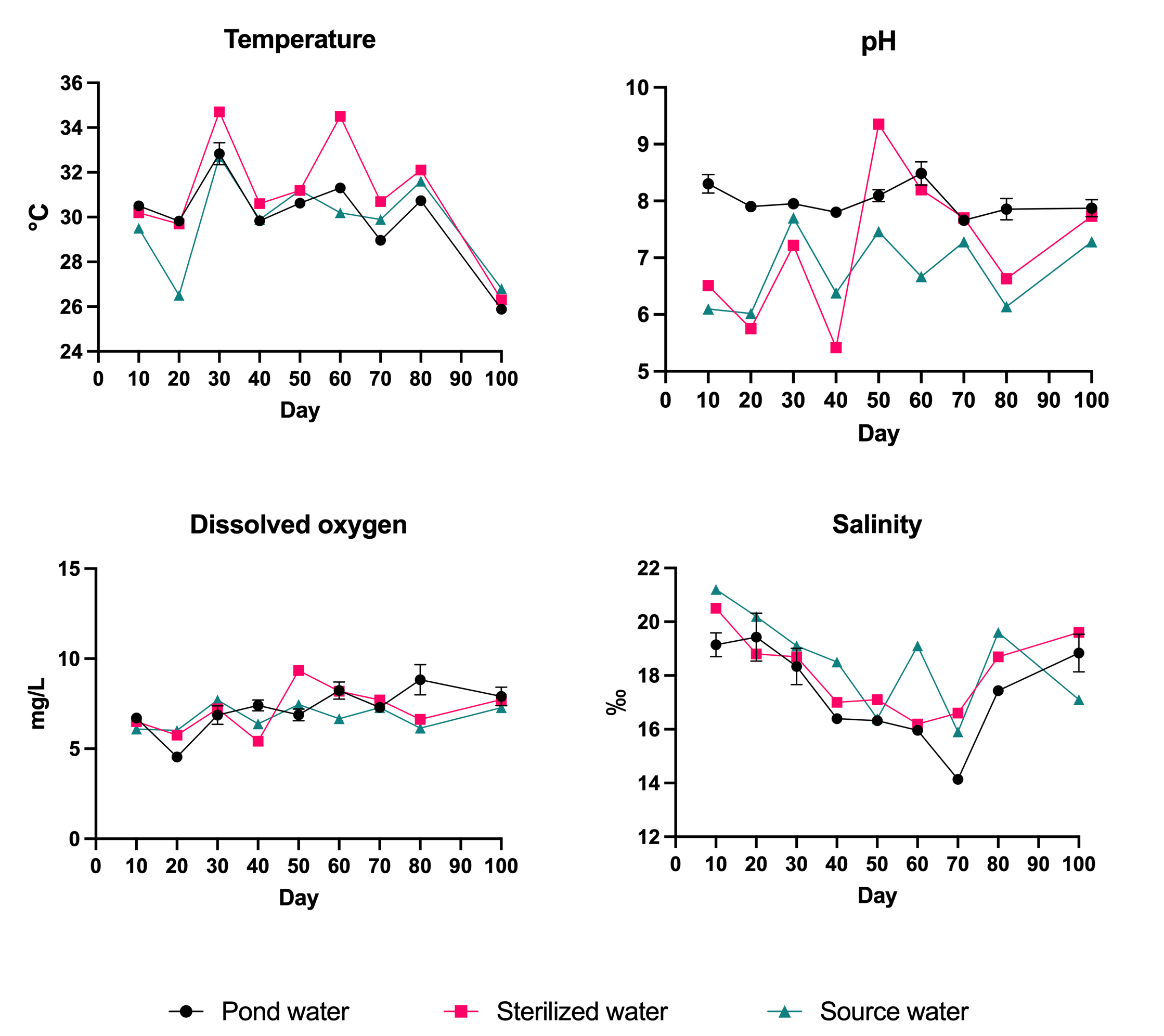


Figure S1


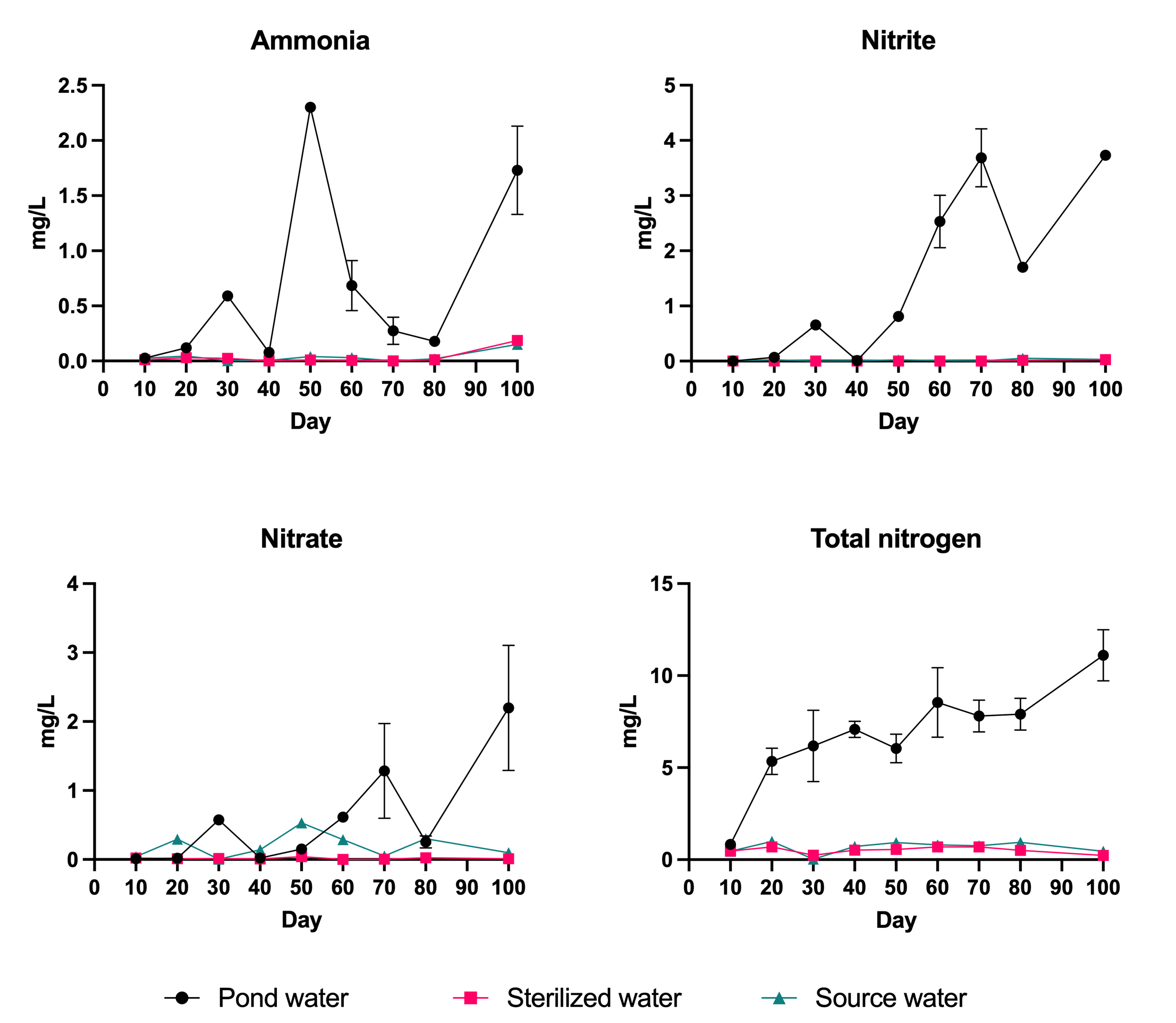


Figure S2


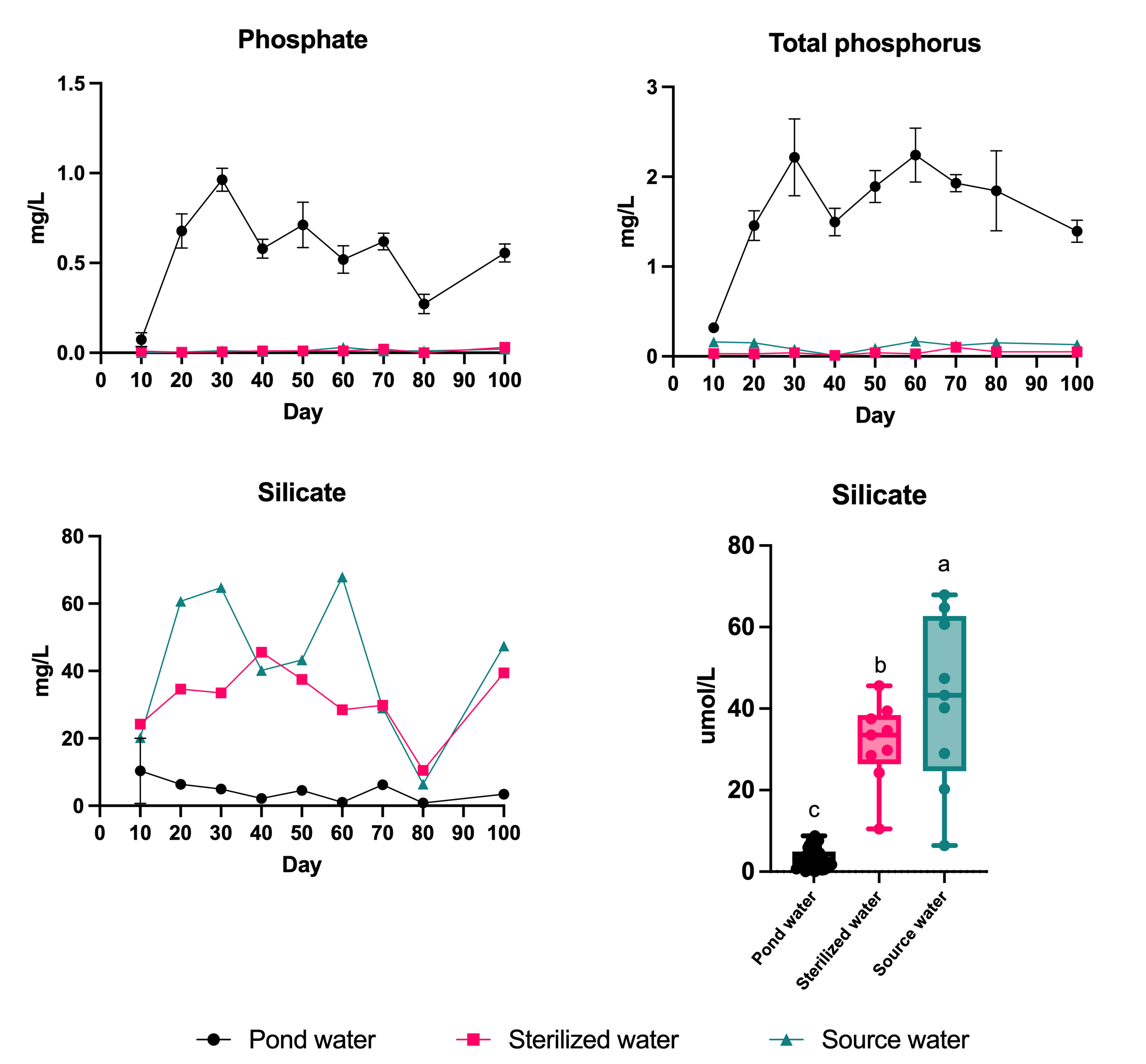


Figure S3


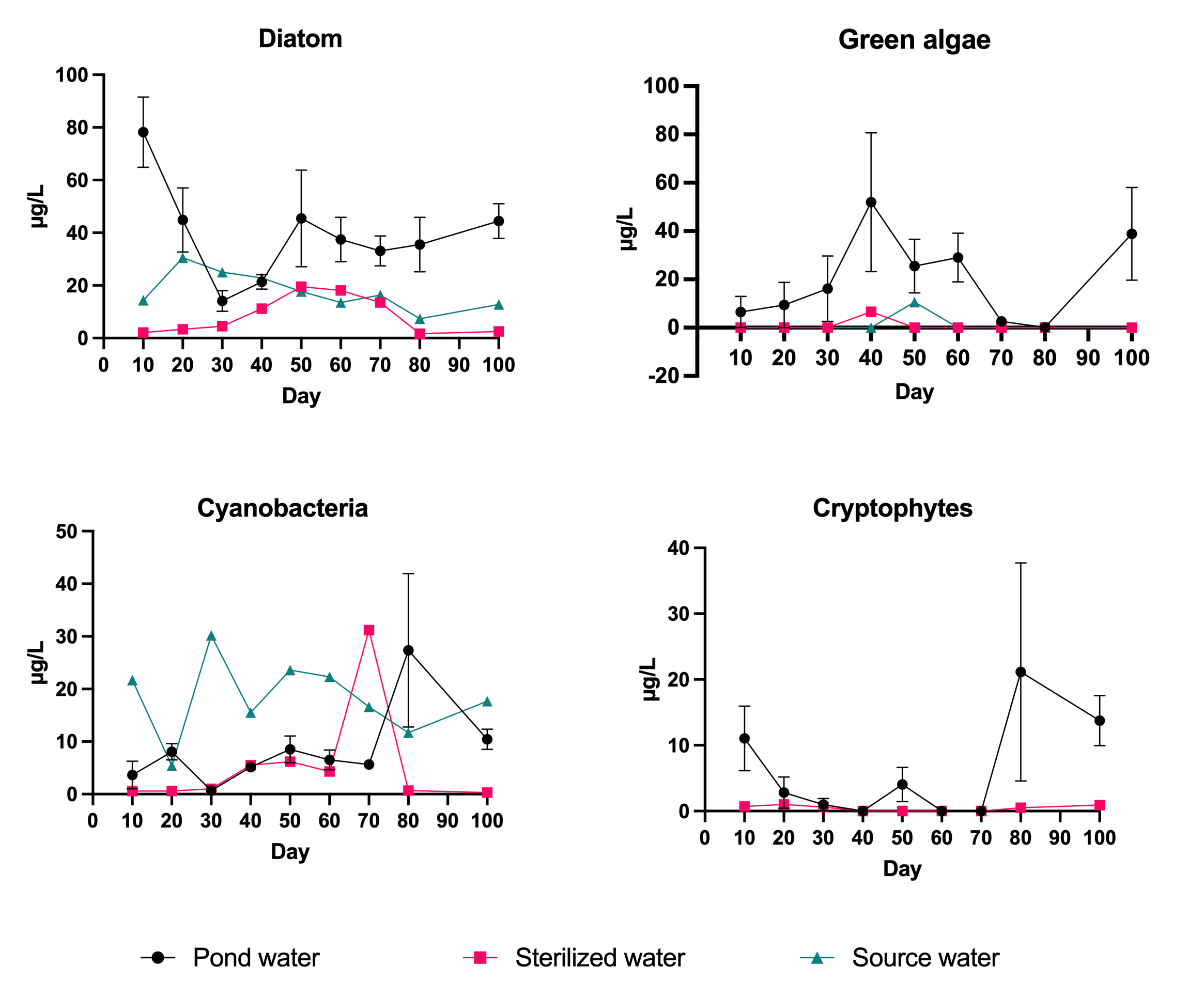


Figure S4


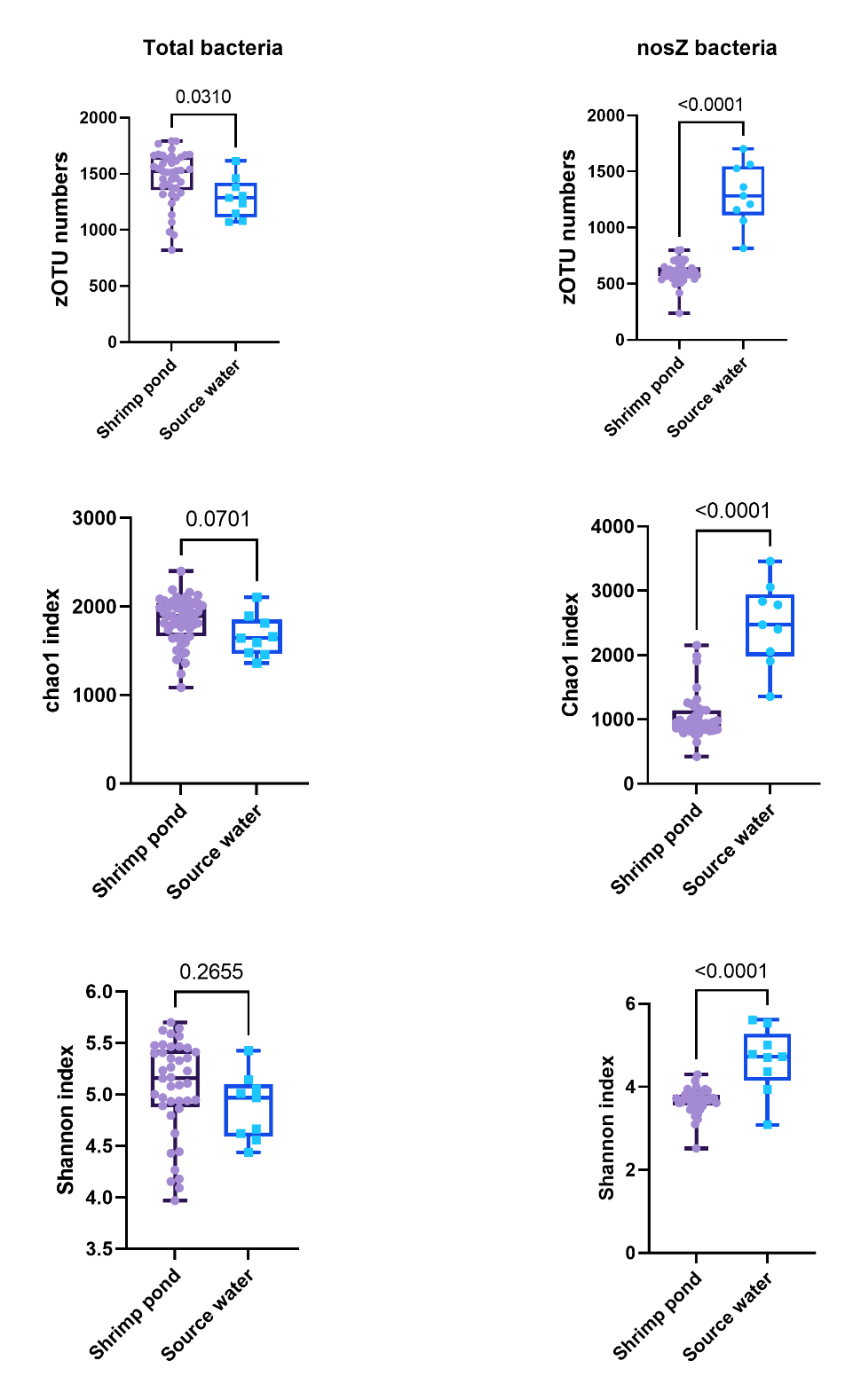


Figure S5


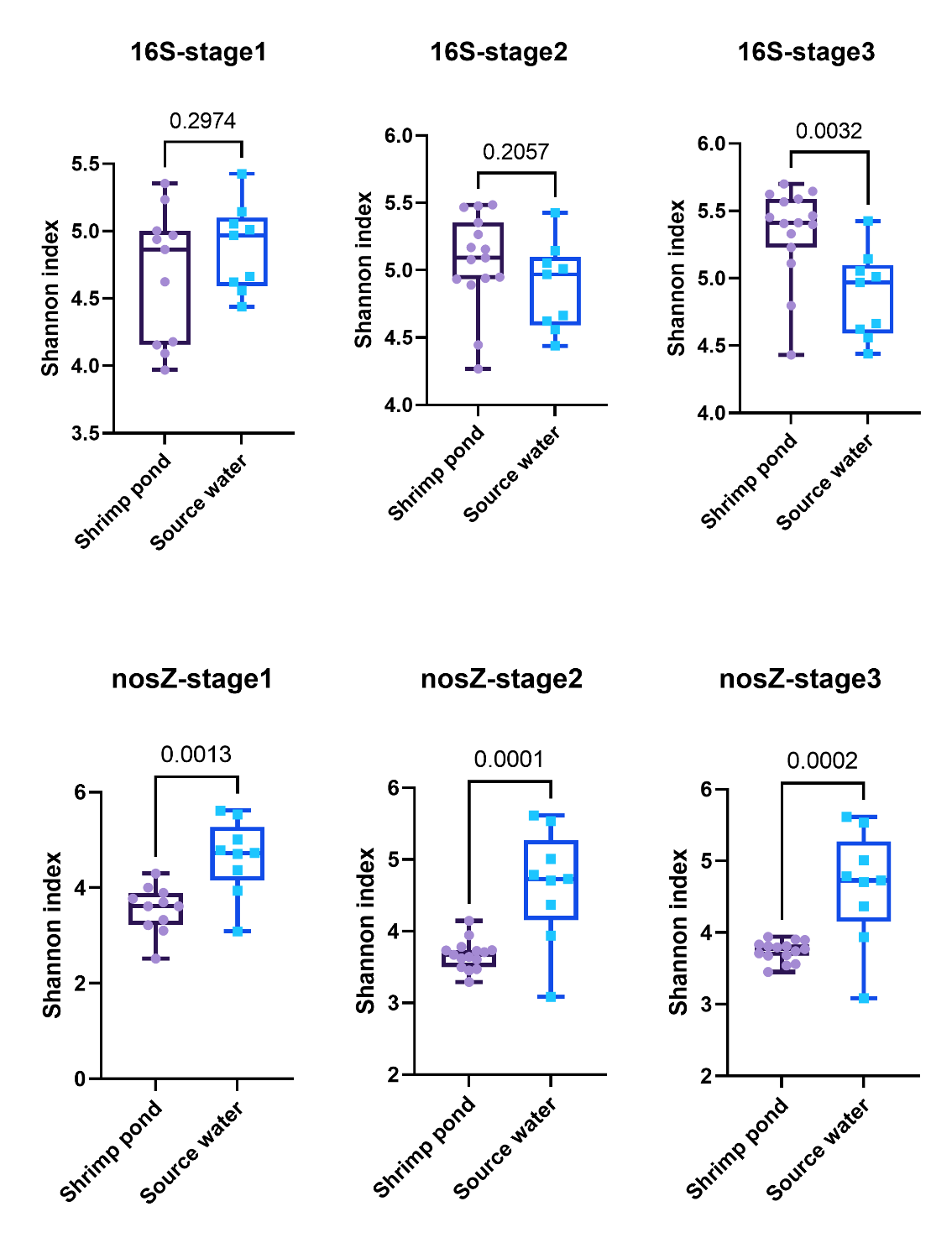


Fig. S6


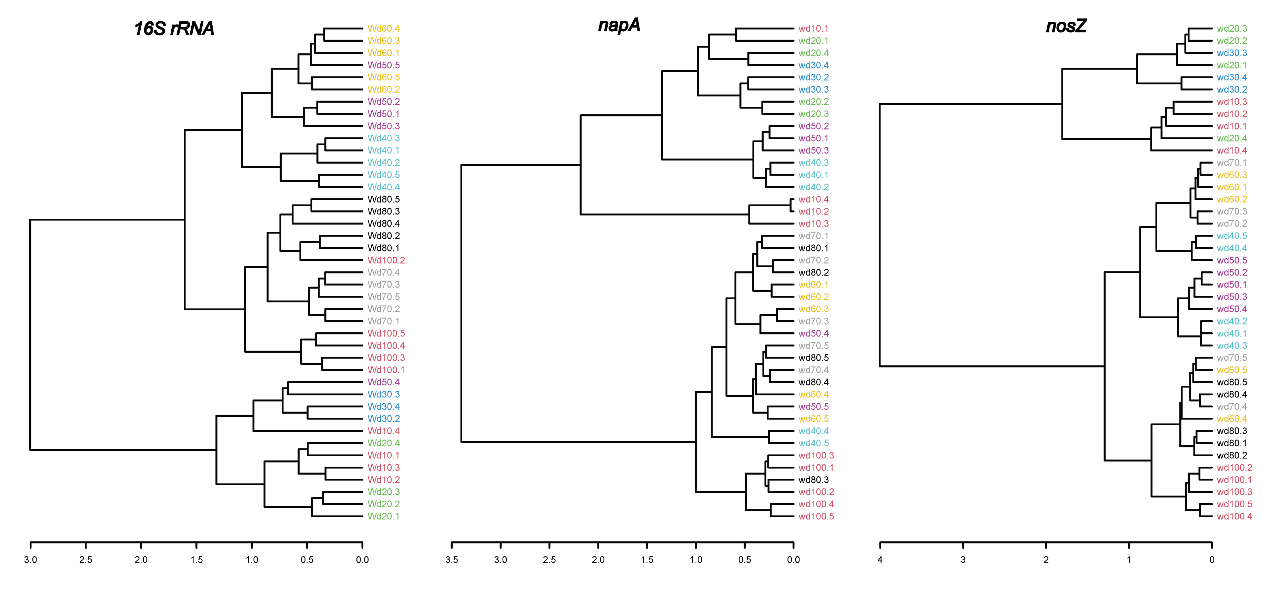


Figure S7


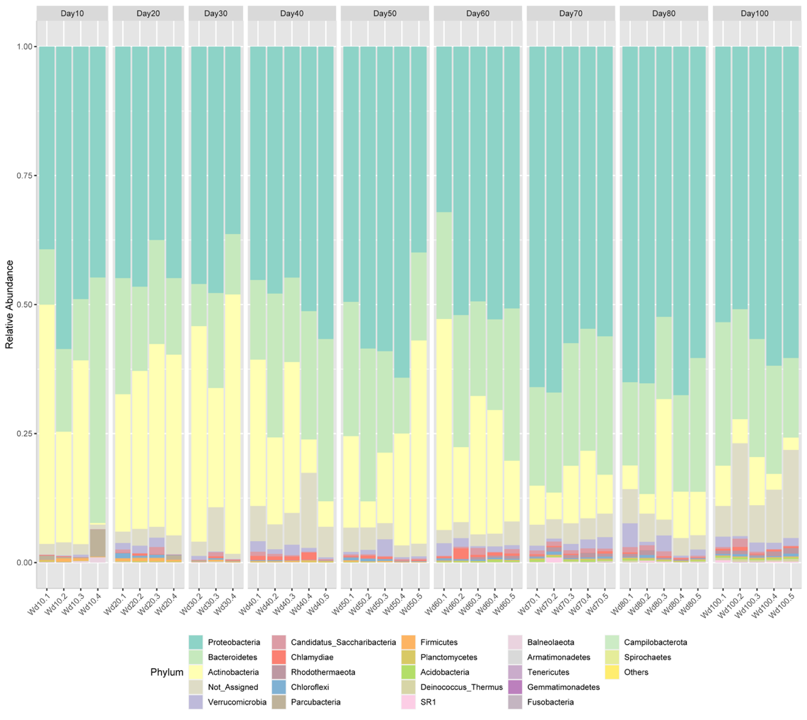


(a)


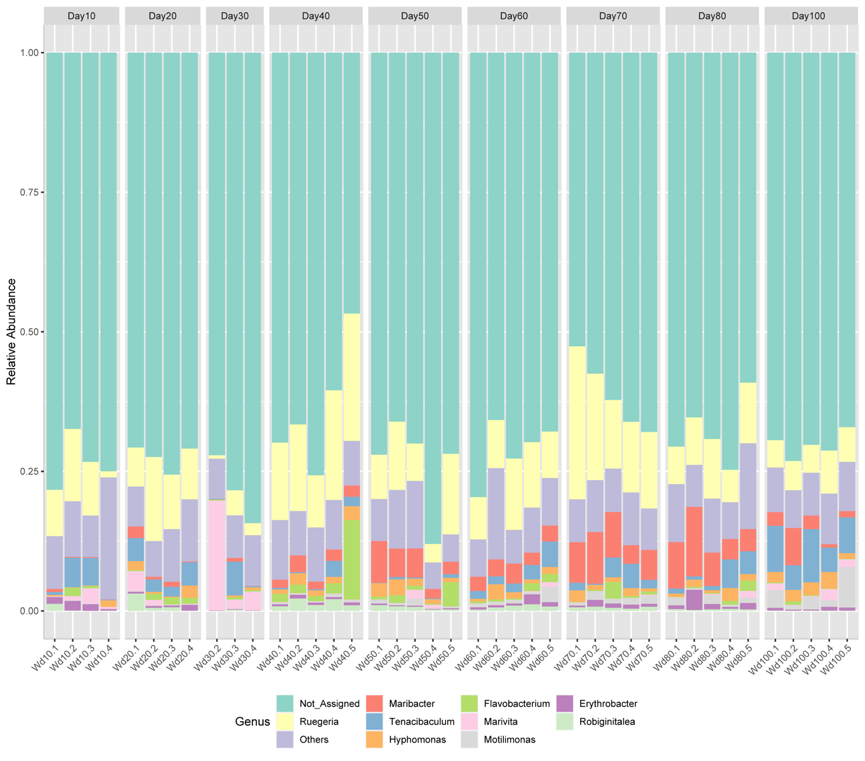


(b)

Figure S8


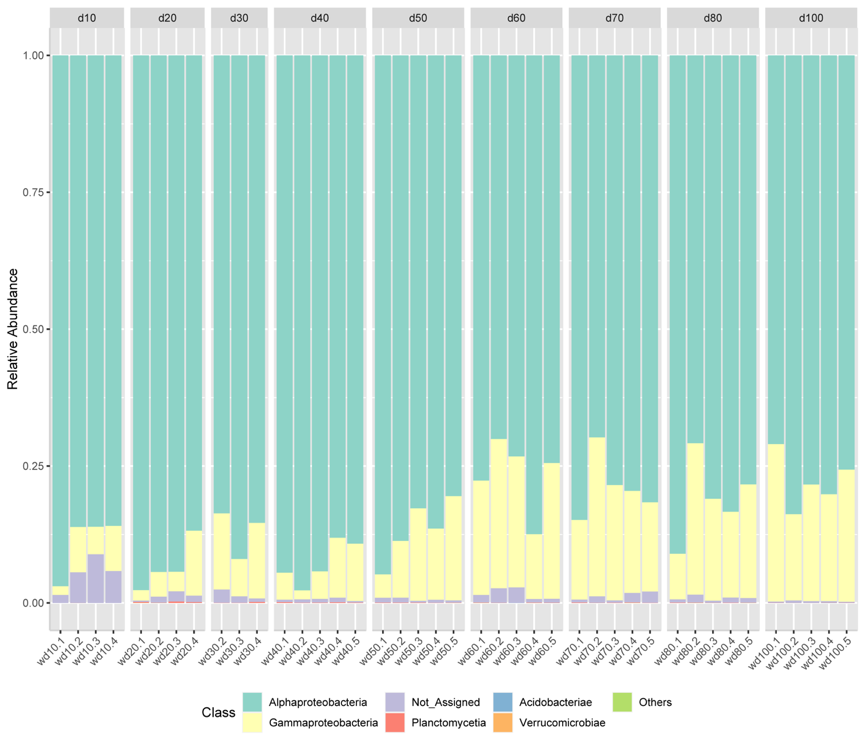


(a)


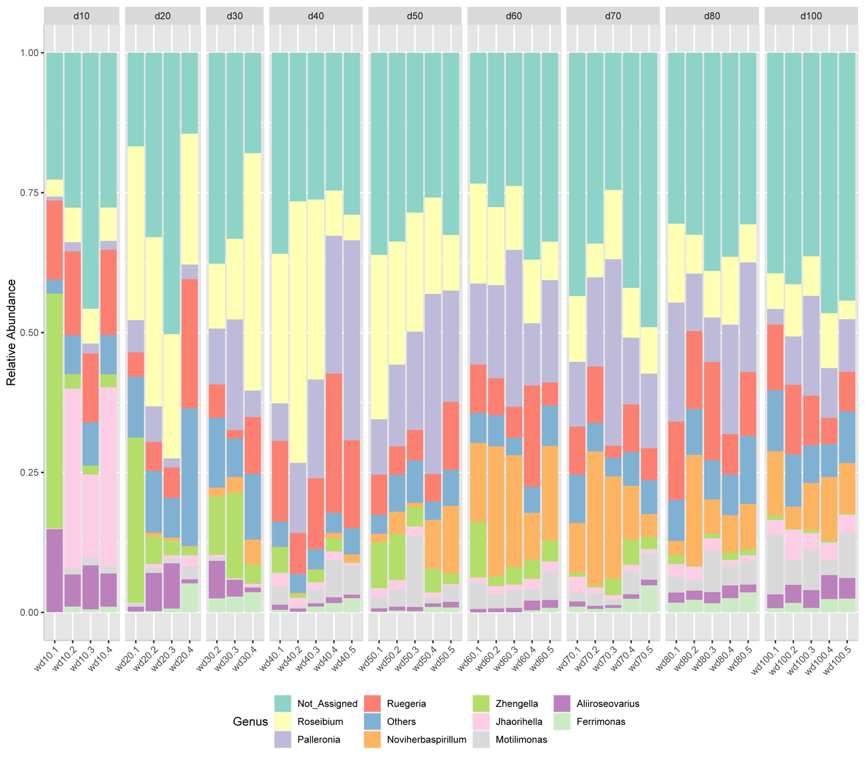


(b)

Figure S9


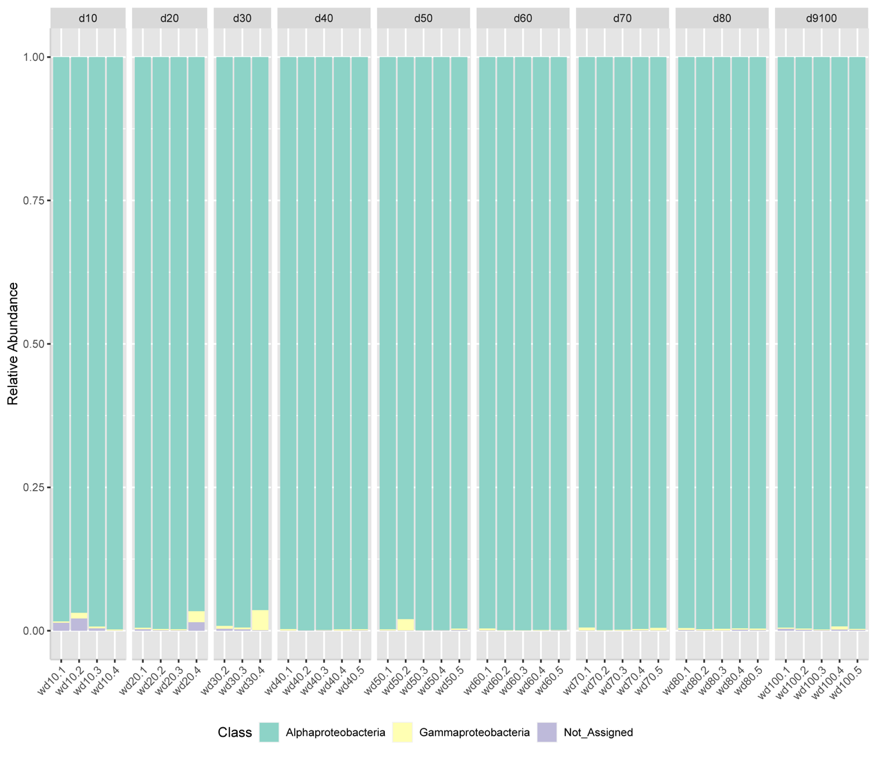


(a)


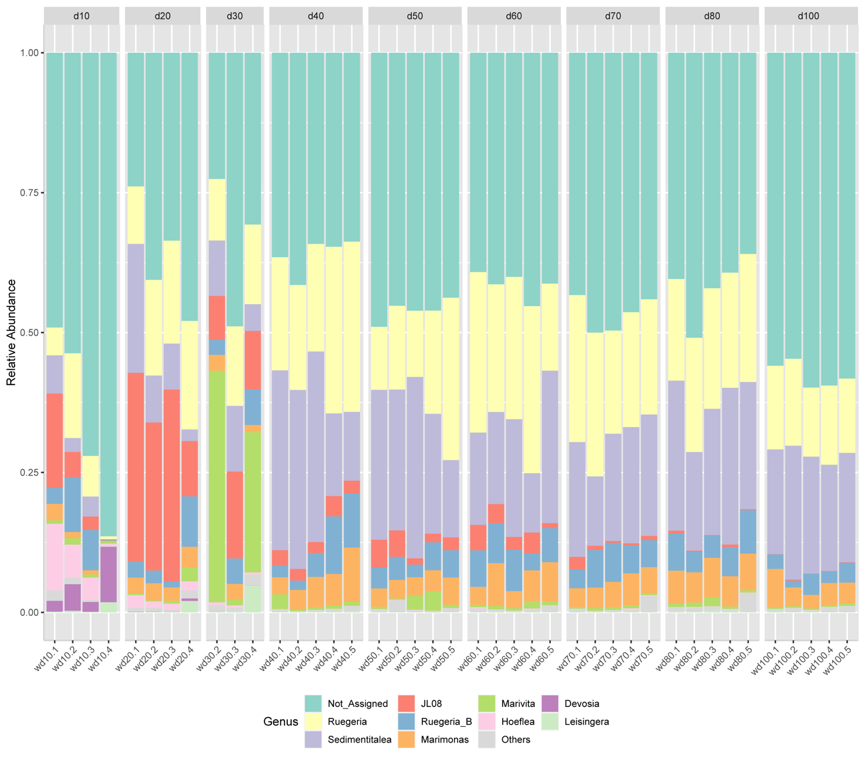


(b)

Figure S10
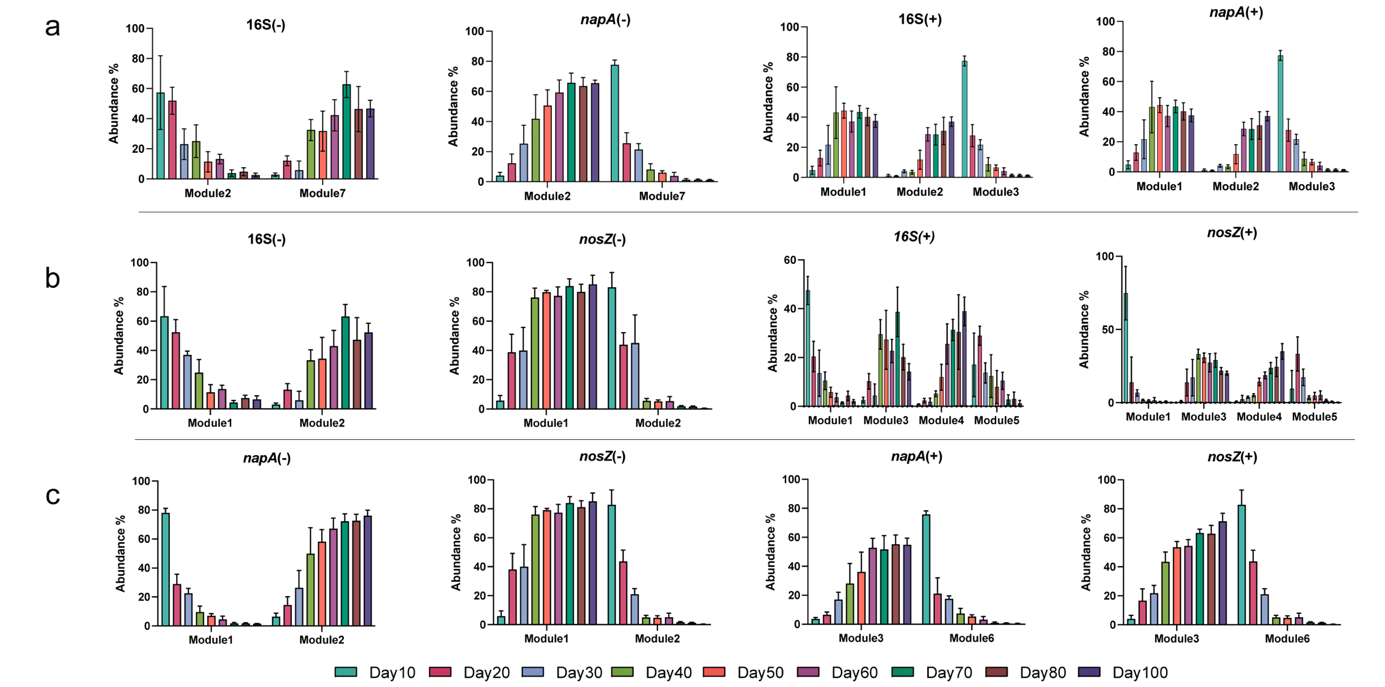


Figure S11


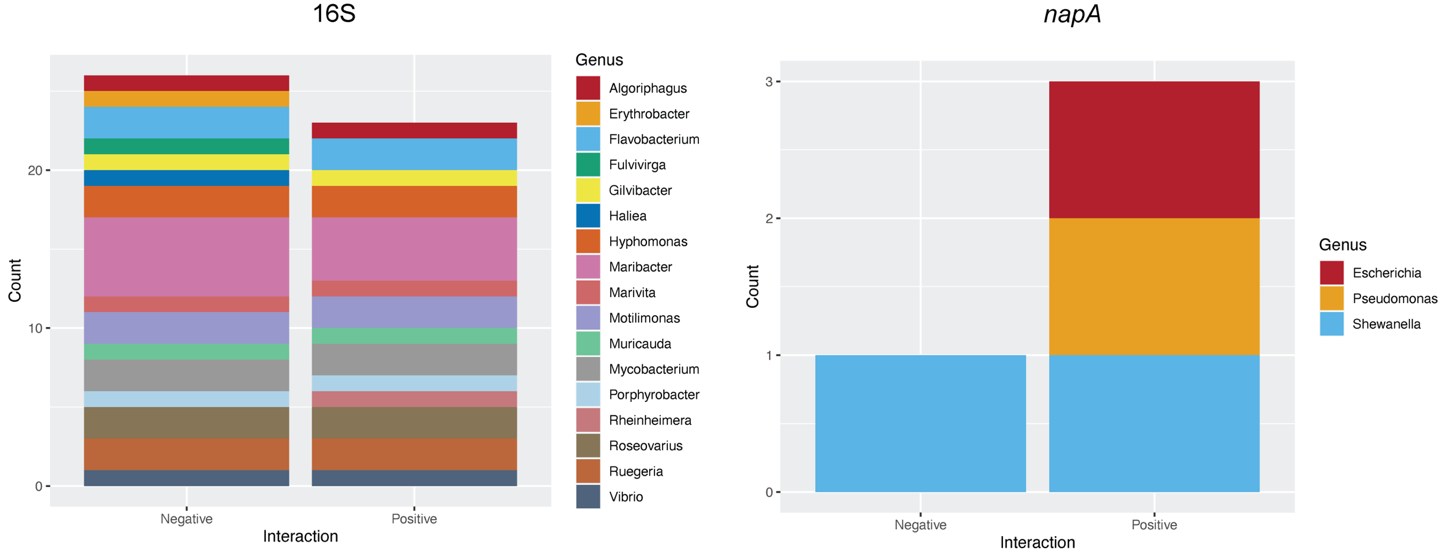


Figure S12


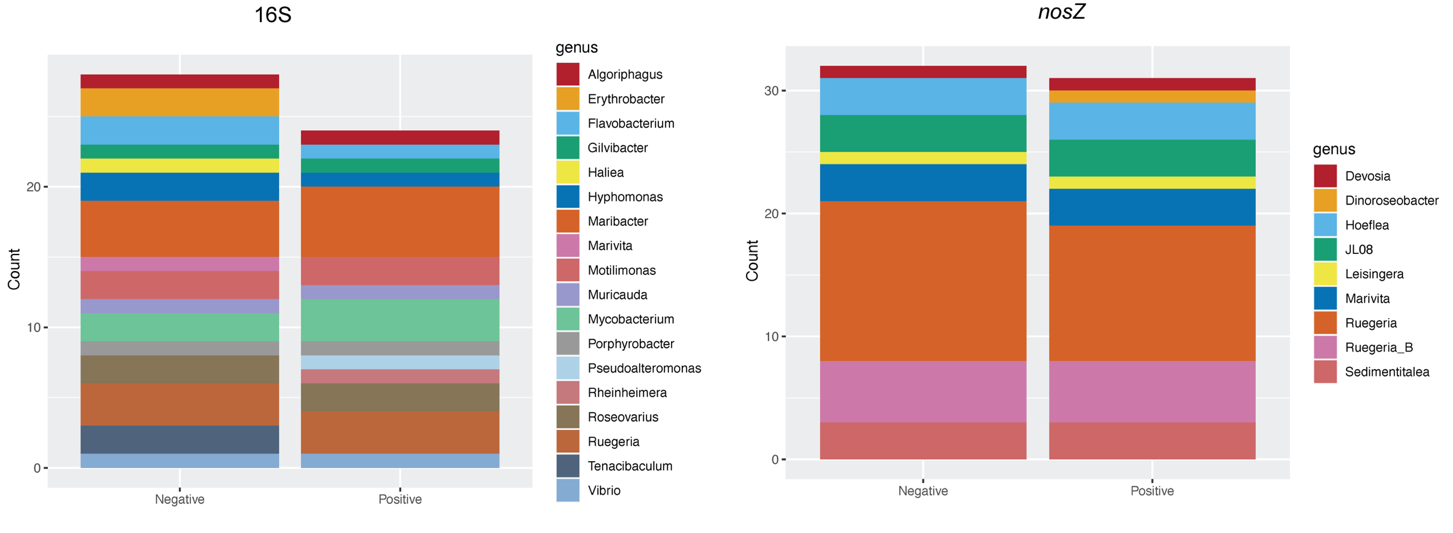


Figure S13


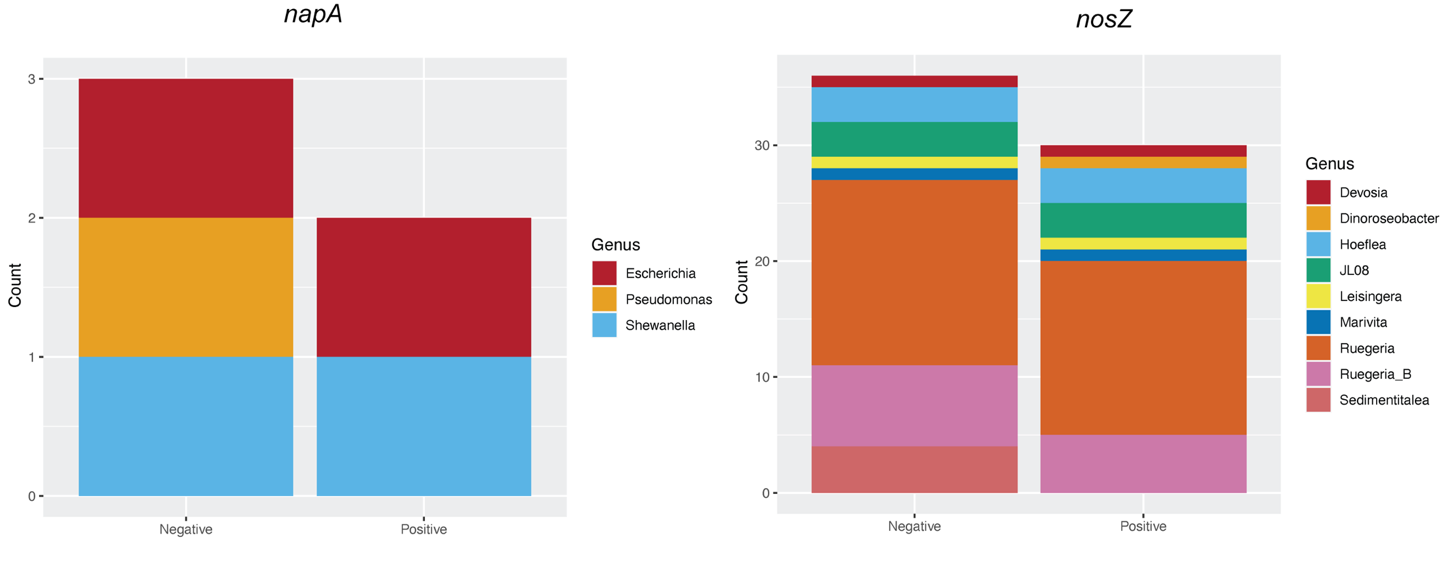


Figure S14


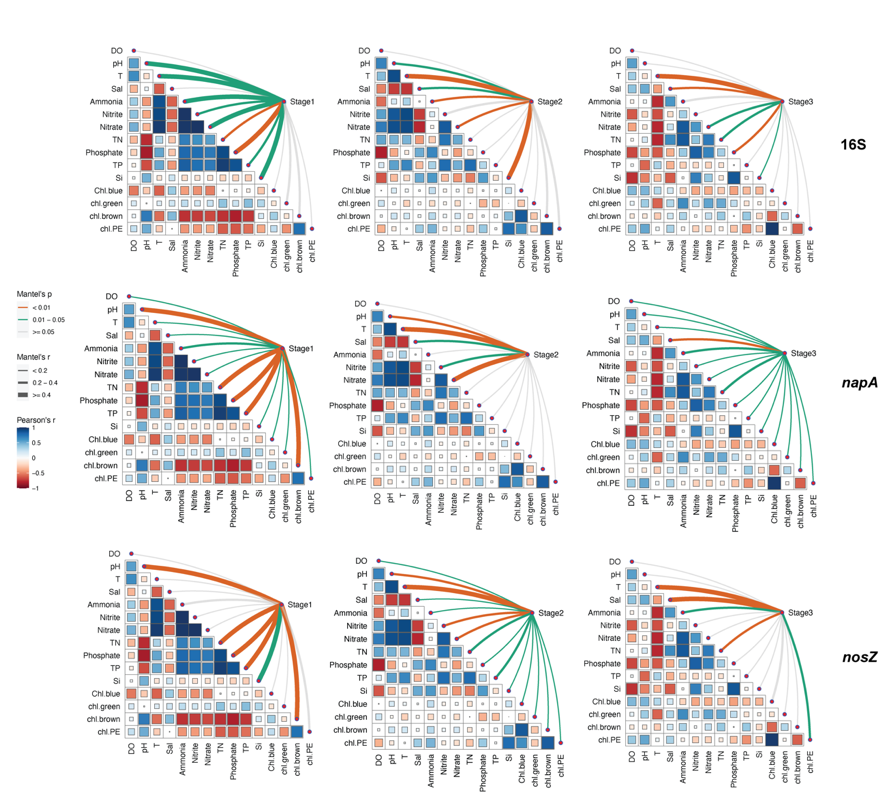


Figure S15
